# eSPC: an online data-analysis platform for molecular biophysics

**DOI:** 10.1107/S2059798321008998

**Published:** 2021-09-24

**Authors:** Osvaldo Burastero, Stephan Niebling, Lucas A. Defelipe, Christian Günther, Angelica Struve, Maria M. Garcia Alai

**Affiliations:** a Departamento de Química Biológica, Facultad de Ciencias Exactas y Naturales, Universidad de Buenos Aires, Intendente Güiraldes 2620, Ciudad Autónoma de Buenos Aires, Argentina; b IQUIBICEN–UBA/CONICET, Intendente Güiraldes 2620, Ciudad Autónoma de Buenos Aires, Argentina; c European Molecular Biology Laboratory, EMBL Hamburg, Notkestrasse 85, 22607 Hamburg, Germany; d Centre for Structural Systems Biology, Notkestrasse 85, 22607 Hamburg, Germany

**Keywords:** eSPC, online servers, open science, molecular interactions, binding affinity, *K*_d_, microscale thermophoresis, differential scanning fluorimetry, protein stability, *T*_m_, ligand screening, molecular biophysics

## Abstract

All biological processes rely on the formation of protein–ligand, protein–peptide and protein–protein complexes. Studying the affinity, kinetics and thermodynamics of binding between these pairs is critical for understanding basic cellular mechanisms. Many different technologies have been designed for probing interactions between biomolecules, each based on measuring different signals (fluorescence, heat, thermophoresis, scattering and interference, among others). Evaluation of the data from binding experiments and their fitting is an essential step towards the quantification of binding affinities. Here, user-friendly online tools to analyze biophysical data from steady-state fluorescence spectroscopy, microscale thermophoresis and differential scanning fluorimetry experiments are presented. The modules of the data-analysis platform (https://spc.embl-hamburg.de/) contain classical thermodynamic models and clear user guidelines for the determination of equilibrium dissociation constants (*K*_d_) and thermal unfolding parameters such as melting temperatures (*T*_m_).

## Abstract 2

eSPC is an online tool to analyze biophysical data from fluorescence, microscale thermophoresis and differential scanning fluorimetry experiments. The modules from the data-analysis platform contain classical thermodynamic models and clear user guidelines for the determination of dissociation constants (*K*_d_) and thermal unfolding parameters such as melting temperatures (*T*_m_).

## 1. Introduction   

Investigating the interactions between biomolecules *in vitro* is a critical step for understanding cellular mechanisms such as signal transduction, small-molecule transportation and substrate modification, and for developing drug and antibody therapies. Biophysical techniques to probe biomolecular interactions include bio-layer interferometry (BLI), isothermal titration calorimetry (ITC), surface plasmon resonance (SPR), fluorescence spectroscopy, microscale thermophoresis (MST) and differential scanning fluorimetry (DSF), also known as fluorescence thermal shift assay (FTSA) or ThermoFluor (Sultana & Lee, 2015 ▸; Velázquez-Campoy *et al.*, 2004 ▸; Myszka & Rich, 2000 ▸; Sindrewicz *et al.*, 2019 ▸; Scheuermann *et al.*, 2016 ▸; Vivoli *et al.*, 2014 ▸).

In the current work, we show the implementation of a webpage to analyze biophysical data. Users can upload their raw data and extract information from their DSF and MST experiments using three online tools. *MoltenProt* has been designed to evaluate the stability of a protein sample (Kotov *et al.*, 2021 ▸) and *FoldAffinity* to determine equilibrium dissociation constants (*K*_d_) from DSF experiments (Niebling *et al.*, 2021 ▸). Both are now available in our EMBL Sample Preparation and Characterization (eSPC) online data-analysis platform. Finally, we present a novel tool called *ThermoAffinity* for the determination of *K*_d_ values from MST and fluorescence spectroscopy experiments. In the current article, we are launching this new platform to be accessible to the whole research community.

Briefly, a DSF experiment consists of measuring the fluorescence emission as a function of temperature. If a change in the signal is detected, information about the protein de­naturation process can be extracted. The fluorescence can be emitted by intrinsic tryptophans (and tyrosines) or by a dye that binds to hydrophobic patches on the protein as it unfolds (for example SYPRO Orange; Niesen *et al.*, 2007 ▸). In both cases, the underlying principle is that modifications in the fluorophore environment translate into changes in the signal. Because DSF allows the stability of a protein to be obtained under different experimental conditions, it can be used to identify optimal detergent conditions for membrane-protein stabilization or to screen protein ligands that stabilize the folded state (Kotov *et al.*, 2019 ▸; Vivoli *et al.*, 2014 ▸; Boivin *et al.*, 2013 ▸). Indeed, the simplicity and high-throughput workflow of the DSF assay, in addition to the low sample consumption, has made this approach ideal for screening and drug-discovery campaigns (Niedziela-Majka *et al.*, 2015 ▸; Seabrook & Newman, 2013 ▸; Mahendrarajah *et al.*, 2011 ▸; Carver *et al.*, 2005 ▸). With regard to binding-affinity estimations from fluorescence-based melting curves, *FoldAffinity*, based on Python scripts released by Bai *et al.* (2019 ▸) and further developed by Niebling *et al.* (2021 ▸), is the first online tool available.

The MST experiment also consists of measuring intrinsic or dye-based fluorescence emission (Baaske *et al.*, 2010 ▸). However, the reported signal depends on two major factors: the relative fluorescence temperature dependence and the directed movement of molecules in a temperature gradient (thermophoresis), both of which are induced by localized heating by an infrared laser (López-Méndez, Uebel *et al.*, 2021 ▸). The sum of all temperature jump-related effects on the fluorescence signal is generally referred to as a temperature-related intensity change (TRIC). The strong sensitivity of this technique to variations in molecular properties such as size, charge, hydration shell and conformation allows biomolecular interactions to be quantified (Scheuermann *et al.*, 2016 ▸; Seidel *et al.*, 2012 ▸). MST can be useful to detect a wide range of events such as the binding of small molecules to proteins, of substrates to enzymes (if the reaction does not proceed) or of ligands to liposomes. In contrast to ITC or SPR, it requires low sample consumption and no surface immobilization. A more detailed explanation of the MST technique can be found in Jerabek-Willemsen *et al.* (2014 ▸) and López-Méndez, Uebel *et al.* (2021 ▸). To our knowledge, *ThermoAffinity* is the first free online resource to analyze MST data.

## 2. Results and discussion   

### 2.1. eSPC description   

**Figure 1:**
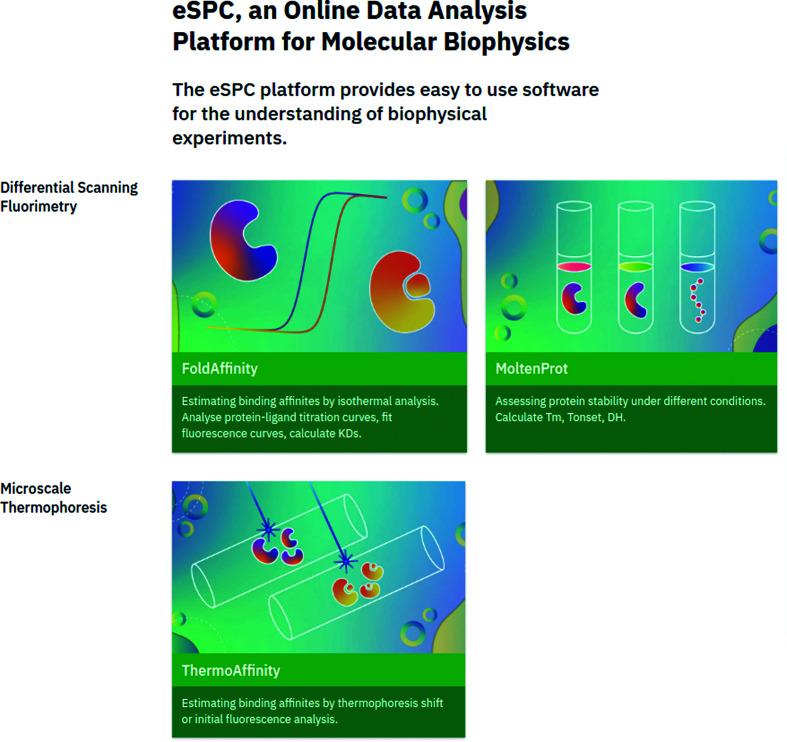
Layout of the eSPC portal with its three online modules: *FoldAffinity*, *MoltenProt* and *ThermoAffinity*.

eSPC is available at https://spc.embl-hamburg.de/ and so far comprises three modules: *MoltenProt* for protein stability studies, *FoldAffinity* and *ThermoAffinity* for binding affinity studies. Fig. 1 ▸ shows the general layout of the eSPC data-analysis platform introducing the three mentioned modules. The usage of each module follows a similar workflow and is accompanied by a user guide, a tutorial with an example file and an associated video. *MoltenProt* and *FoldAffinity* work with the output file generated by a quantitative PCR (qPCR) instrument or the processed datasheet file generated by the Prometheus instrument (Nanotemper) after a nanoDSF experiment (*MoltenProt* user documentation, Section 1.1). *ThermoAffinity* accepts both MST Monolith instrument (Nanotemper) output files (datasheet) or custom Comma Separated Value (CSV) files (*ThermoAffinity* user documentation, Section 1.1). All modules were developed in a user-focused fashion and allow fast and easy high-quality data analysis with the possibility of exporting publication-grade figures.

### 2.2. *MoltenProt*   

*MoltenProt* is a tool to estimate thermodynamic parameters from melting curves that was recently developed as a desktop application (Kotov *et al.*, 2021 ▸). An example of a practical implementation of this module would be the screening of integral membrane-protein (IMP) stability in detergent or membrane-like environments (Kotov *et al.*, 2019 ▸). Monitoring the unfolding process of an IMP under different experimental conditions is critical and may determine the success of a structural biology study.

**Figure 2:**
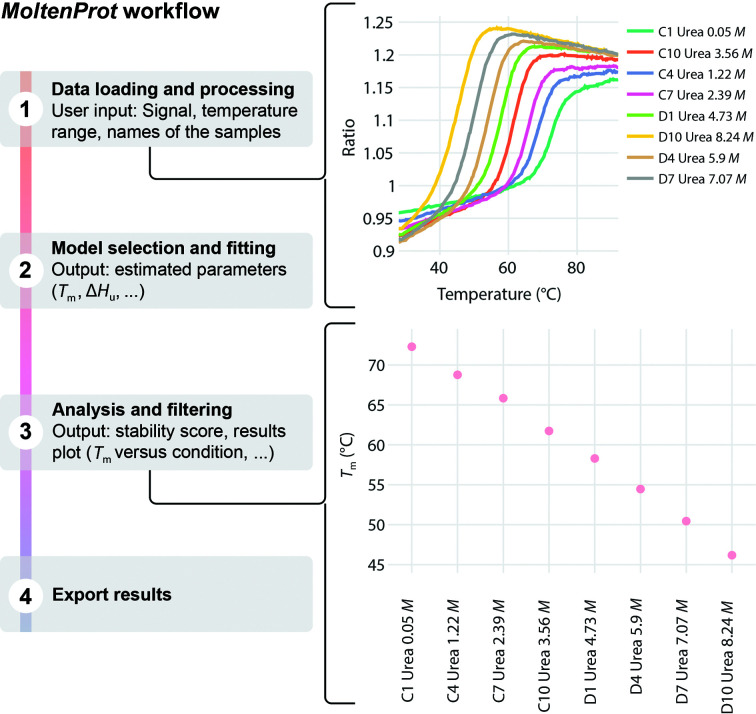
The *MoltenProt* pipeline consists of four steps. Firstly, the data are loaded and preprocessed by selecting the temperature range, applying smoothening or normalization transformations and selecting the experimental conditions to analyze. In this step, the user will see the signal (330 nm, 350 nm, ratio and derivatives) versus temperature plots. Secondly, the melting curves are fitted using one of the five available models. The fitted curves to the raw data and the estimated parameters with their respective errors are presented. As an example, a reversible two-state model will yield the enthalpy of unfolding Δ*H*_u_ and the melting temperature *T*_m_. Thirdly, the fitted models are used to obtain a protein stability score or to visualize different plots such as ‘unfolded fraction versus temperature’ or ‘*T*_m_ versus experimental condition’. The fitted curves can be filtered according to certain criteria such as low relative errors in the estimated parameters. Fourthly, a complete report of the analysis can be exported together with individual CSV files containing the model-derived information.

Briefly, the pipeline of the online version of *MoltenProt* consists of four steps (Fig. 2 ▸). Firstly, the data are loaded and preprocessed. At this point, the user can select the signal and the temperature range of interest and, if necessary, to smooth or normalize the data. If the input file originates from a DSF experiment performed in a qPCR instrument, the only available signal will correspond to the fluorescence of a hydrophobic dye (Niesen *et al.*, 2007 ▸). In the case of a DSF experiment, *MoltenProt* can analyze the intrinsic fluorescence of the protein [measured at 330 nm (*F*_330_) or 350 nm (*F*_350_)], the fluorescence ratio (*F*_350/330_) or the scattering signal (*MoltenProt* user documentation, Section 1.1). Special caution must be taken when selecting the signal to be analysed. As an example, it has been suggested that the fluorescence ratio would not be a reliable parameter for the evaluation of unfolding transitions in particular cases (Žoldák *et al.*, 2017 ▸). One of five models can then be selected to fit the melting curves. We have implemented three thermodynamic-based models that include a two-state reversible or irreversible model and a three-state model with a short-lived intermediate. The other two models are empirical versions of the two-state and three-state reversible models. Typically, one would apply an equilibrium (reversible) two-state unfolding model and estimate the melting temperature *T*_m_ (defined as the temperature at which half of the protein is unfolded) and enthalpy of unfolding Δ*H*_u_. These two parameters are then used to estimate the fraction of unfolded protein at all of the measured temperatures and the *T*_onset_ (defined as the temperature at which 1% of the molecules are unfolded). To assess the robustness of the fitting, we provide the relative errors of the fitting parameters and residuals. Thirdly, the result of the fitting is analyzed and filtered according to certain criteria. As an example, the user can remove fitted curves where the estimated parameters have relative errors larger than 50% or are too close to their fitting boundaries. In this step, the user can decide which sample is the optimal sample by comparing the free energy of unfolding at 25°C, the melting temperature or even the unfolded fraction versus temperature curves. Finally, the model-derived information can be exported to CSV files and a complete report can be generated.

### 2.3. *FoldAffinity*   

DSF is often used in drug-discovery campaigns as a tool to probe the interaction between compounds and a protein of interest (Amaning *et al.*, 2013 ▸). After a hit (a ligand that stabilizes the protein) is found and confirmed by an orthogonal assay, researchers may wish to crystallize the complex to gain insight into the molecular interactions at an atomistic level and reveal future paths for ligand optimization. Deciding which hit to focus on notably depends on the affinity of the interaction. To address this issue, *FoldAffinit*y allows the user to perform an isothermal analysis approach to quantify the binding affinity. The analysis is based on a thermodynamic model that comprises two fitting steps (Bai *et al.*, 2019 ▸; Niebling *et al.*, 2021 ▸). Firstly, the fluorescence-based melting curves are adjusted to an unfolding model to determine the unfolded fraction as a function of temperature and then, at a fixed temperature, the unfolded fraction versus ligand concentration curve is generated to estimate the binding affinity. The equations involved are provided in the supporting information.

**Figure 3:**
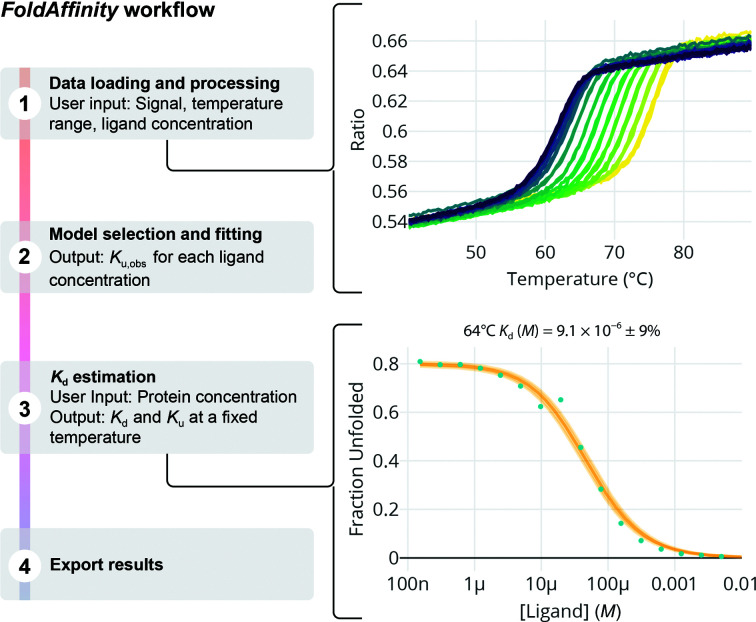
The *FoldAffinity* pipeline can be divided into four steps. In the first step, the data are loaded and preprocessed. The preprocessing includes selecting the temperature range, smoothening the data and adding information about the ligand concentration of each capillary/well. The signal versus temperature plot will be colour-coded using a base-10 log scale and the viridis palette. Secondly, each melting curve is fitted to a two-state unfolding model that implicitly takes ligand binding into account. In this step, the user can see the fitted curves to the raw fluorescence data and identify temperature regions that deviate from the model. Thirdly, the unfolded fraction obtained from the previous fitting (*K*_u,obs_) is used in combination with the protein concentration to estimate the binding affinity (*K*_d_) and the unfolding constant (*K*_u_) at a fixed chosen temperature (64°C in the example shown). Finally, individual CSV files with the model-derived information can be exported.

The fitting procedure of *FoldAffinity* consists of three steps (Fig. 3 ▸). Firstly, the data are loaded and preprocessed (*FoldAffinity* user documentation, Section 2.1). At this point, the user can select the signal and the temperature range of interest, smooth the data to remove spikes if necessary and include information about the ligand concentration at each position (capillary/well). As in *MoltenProt*, the signal can be produced by intrinsic protein fluorescence from tryptophans/tyrosines or by a hydrophobic dye (Niesen *et al.*, 2007 ▸). Each preprocessed thermal curve is then fitted to a reversible two-state unfolding model that implicitly takes into account ligand binding. In this way, for each ligand concentration we can express the amount of unfolded fraction as a function of the temperature using the equilibrium constant *K*_u,obs_. This fitting step, which is called ‘Fluorescence fit’ in *FoldAffinity*, can be performed locally for each curve or in a global fashion by constraining all fittings to have the same value for some of the parameters. Thirdly, at a fixed temperature, which is typically chosen close to the melting temperature of the free protein, the amount of unfolded fraction versus ligand concentration curve is used to estimate the equilibrium dissociation constant *K*_d_. This temperature can be selected manually by the user in the ‘Fit unfolded fraction’ section of *FoldAffinity* to maximize the dynamic range of the unfolded fraction (*y* axis) and minimize the error in the estimation of the equilibrium dissociation constant. We have implemented and thoroughly tested a 1:1 binding model and have also included the equations to fit a 1:2 binding model.

Additionally to the isothermal analysis, in *FoldAffinity* we allow the fitting of a thermodynamic-based model to estimate the binding affinities directly from the observed melting temperatures (Yoshida *et al.*, 2019 ▸; Schellman, 1975 ▸; Supplementary Fig. S1). This last model works well and is simpler to use if certain conditions are satisfied, such as assuming that the free ligand concentration equals the total ligand concentration and that the ligand is completely soluble at the observed melting temperatures.

### 2.4. *ThermoAffinity*   

**Figure 4:**
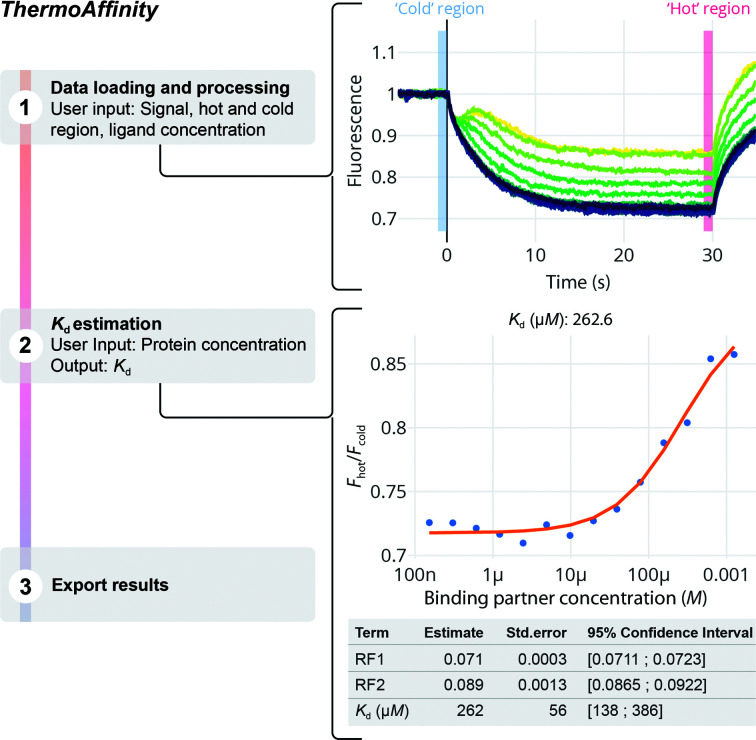
The *ThermoAffinity* pipeline consists of three steps. In the first step, the data are loaded and preprocessed. The preprocessing includes smoothening or normalizing the data, adding information about the ligand concentration and selecting the ‘hot’ and ‘cold’ regions to obtain the normalized fluorescence *F*_norm_ = *F*_hot_/*F*_cold_. The fluorescence signal versus time plot will be colour-coded using a base-10 log scale and the viridis palette. Secondly, the thermophoresis signal versus binding partner concentration curve, together with the protein concentration, is used to obtain the binding affinity (*K*_d_) and the numeric values RF1 and RF2 (the fluorescence signal of 1 µ*M* unbound protein and 1 µ*M* complexed protein, respectively). All of the estimated parameters are presented with their respective errors and associated confidence intervals. Finally, individual CSV files with the model-derived information can be exported.

The MST technology can quantify interactions with almost no limitations with respect to molecule size or molecular weight. It allows the determination of a wide range of binding affinities (from low-millimolar to picomolar), it requires very low amounts of sample, has been shown to work with plenty of different buffers including cell lysates (Jerabek-Willemsen *et al.*, 2014 ▸) and has been used in drug-discovery workflows (Rainard *et al.*, 2018 ▸; Linke *et al.*, 2016 ▸). During an MST experiment, a focused infrared laser locally heats a defined sample volume and the change in the fluorescence signal induced by the temperature gradient is detected. During the ‘cold’ region of the experiment (*i.e.* before the laser-induced heating), the molecules are homogeneously distributed and the fluorescence signal (tryptophan or fluorophore-labelled protein) is constant all along the capillary (Fig. 4 ▸). Immediately after the activation of the IR laser (‘hot’ region), the temperature abruptly changes (*T*-jump) and, since the initial heating occurs much faster than diffusion, the signal changes are mainly due to the fluorescence temperature dependence of the fluoro­phore. Upon generation of the thermal gradient, the thermophoretic effect also contributes to the signal. Typically, the fluorescence change is measured for around 20–30 s and after deactivation of the infrared laser an inverse jump in the signal occurs. If binding produces a change in the overall signal, equilibrium dissociation constants can be readily estimated from ligand-titration curves. For this analysis, the change in the normalized fluorescence (*F*_norm_), which is usually calculated as the quotient *F*_hot_/*F*_cold_, is used, where *F*_hot_ and *F*_cold_ are the mean fluorescence signals of the ‘hot’ and ‘cold’ regions, respectively. It has been suggested that analysing the signal right after thermal perturbation (within 1–2 s of the *T*-jump) leads to fewer thermally induced artefacts (López-Méndez, Uebel *et al.*, 2021 ▸).

In *ThermoAffinity*, the user can load the output file of an MST experiment and easily estimate the binding affinities (*ThermoAffinity* user documentation, Section 1.1). Two steps are required. Firstly, the data are loaded, the ‘hot’ and ‘cold’ regions are selected to optimize the signal-to-noise ratio, and the ligand concentration information is included. Before continuing with the analysis, the user should verify that the initial fluorescence signal does not change significantly in the presence of the ligand (±20% relative to the average signal). The user must then select between the available models that include 1:1 or 1:2 binding and fit the *F*_norm_ versus ligand concentration curve. The equilibrium dissociation constant *K*_d_ and the accompanying parameters of the model are shown together with the errors and the 95% confidence interval (Fig. 4 ▸).

*ThermoAffinity* was developed to analyze MST experiments, but in practice it can be used to estimate binding affinities from the specific intrinsic tryptophan quenching or enhancement produced by ligand binding as a linear combination of the signal produced by the complex and the unbound state. As a consequence, custom CSV files with two columns, signal and ligand concentration, can be loaded for analysis in *ThermoAffinity*.

### 2.5. Case study: sample optimization and binding affinity estimation using protein kinase G (PknG)   

*Mycobacterium tuberculosis*, the causative agent of tuberculosis, has 11 serine/threonine kinases that are involved in the regulation of metabolic processes, gene transcription, cell division and pathogen–host interactions (Prisic & Husson, 2014 ▸). The protein serine/threonine kinase G (PknG) is one of the two cytosolic serine/threonine kinases and is of particular interest due to its essential role in the survival within the host and in the regulation of the carbon and nitrogen metabolisms (Scherr *et al.*, 2007 ▸; Rieck *et al.*, 2017 ▸).

#### 2.5.1. Sample optimization   

Crystallization still mostly remains a trial-and-error technique, and various screening experiments are performed before one or more optimal experimental conditions are found. One way of increasing the success rate is to work with a stabilized sample. Therefore, searching for buffer conditions that increase the global thermal stability is desirable. Indeed, this factor explains why many protein kinases have been crystallized in a holo state (ligand-bound) but there are not so many structures of the apo state (unbound) available, and the case of PknG is a good example (Lisa *et al.*, 2015 ▸; Scherr *et al.*, 2007 ▸). To understand which experimental conditions (de)stabilize PknG, we expressed a smaller construct that comprises the kinase and rubredoxin domains and performed a nanoDSF screening using buffers at different pH values and adding different ligands [Mg^2+^, AX2017, ATPγS, CaCl_2_ and 20%(*w*/*v*) PEG 4000] (Lisa *et al.*, 2015 ▸).

**Figure 5:**
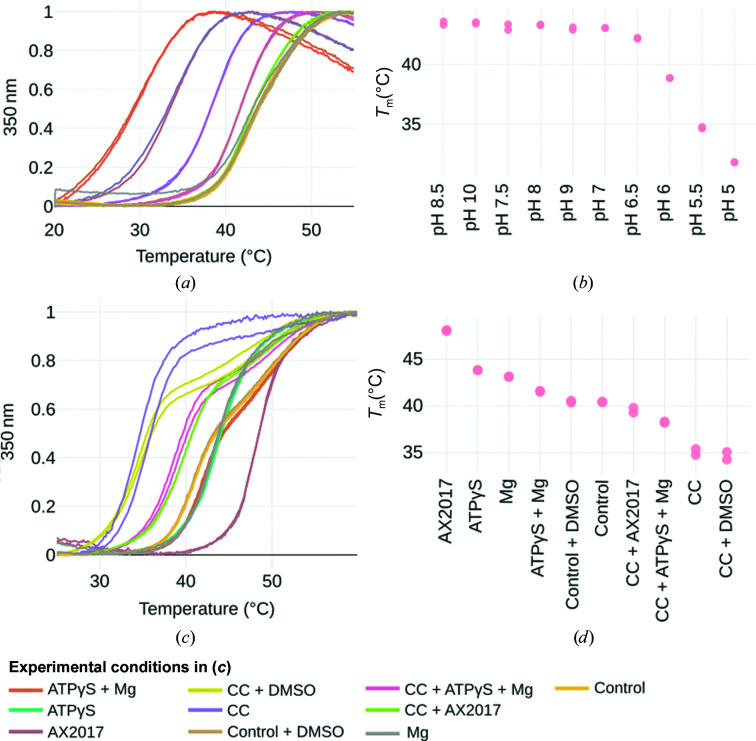
A fluorescence-based thermal shift assay reveals experimental conditions that stabilize PknG. (*a*) Melting curves of PknG at different pH values (after ‘min–max normalization’ in the Load Input step, a transformation that scales the values between 0 and 1 without changing the shape of the curve). (*b*) Melting temperatures (*T*_m_) from the curves in (*a*) estimated using the two-state reversible unfolding model implemented in *MoltenProt*. (*c*) Melting curves (after ‘min–max normalization’) of PknG under different experimental conditions, including the addition of ligands (AX2017 in 1% DMSO, ATPγS and Mg) or a 1:1 mixing of PknG with the crystallization condition (CC) used by Lisa and coworkers to obtain the crystal structure with PDB code 4y12. The complete list of experimental conditions can be found in Section 4.1.4. (*d*) Melting temperatures (*T*_m_) from the curves in (*c*) estimated using the two-state reversible unfolding model implemented in *MoltenProt*.

Regarding the pH screening, the melting curves displayed in Fig. 5 ▸(*a*) suggest a pH-dependence of PknG stability. The melting temperatures *T*_m_, estimated using the two-state equilibrium unfolding model implemented in *MoltenProt*, demonstrate that the stability of PknG is almost constant between pH 10 and 6.5, at which point the stability starts decreasing (Fig. 5 ▸ *b*). Similar trends were observed when analyzing the *T*_onset_ or the unfolded fraction versus temperature curves (Supplementary Figs. S2 and S3). Using the same methodology, we estimated the *T*_m_ values of PknG in the presence of different ligands or mixed with the crystallization condition described by Lisa *et al.* (2015 ▸). The results, presented in Figs. 5 ▸(*c*) and 5 ▸(*d*), suggest a clear stabilization by the known ligand AX2017 (40 µ*M*) and only a slight difference on the addition of ATPγS (100 µ*M*), MgCl_2_ (2 m*M*) or the addition of the ATP analog and MgCl_2_ (200 µ*M* ATPγS and 2 m*M* MgCl). Interestingly, the crystallization condition (CC in Fig. 5 ▸ *d*) decreases the stability of PknG by almost 8°C, but this can be partially reversed by the addition of AX2017 or of ATPγS (200 µ*M*) and MgCl (2 m*M*).

#### 2.5.2. Binding affinity between PknG and AX2017   

Several attempts have been made to find inhibitors of PknG, leading to the discovery of the ligand called AX2017, which showed a half-inhibitory concentration (IC_50_) in the low-micromolar range using full-length PknG (Vivoli, Novak, Littlechild & Harmer, 2014 ▸; Scherr *et al.*, 2007 ▸). The two estimations (IC_50_ of 0.39 and 1.2 µ*M*) were acquired by analyzing (auto)phos­phorylation in an *in vitro* kinase assay with ^32^P-labelled ATP.

**Figure 6:**
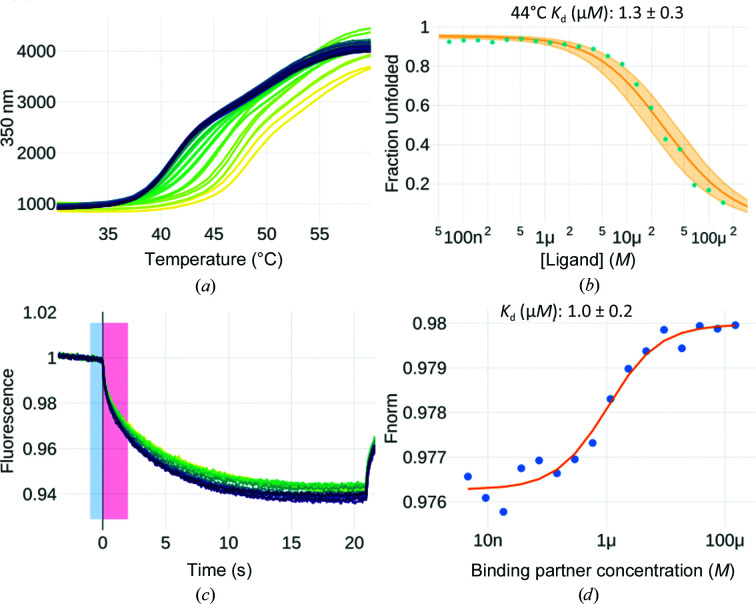
Binding-affinity estimation between PknG and AX2017. (*a*) Fluorescence-based melting curves of PknG mixed with different concentrations of AX2017. At 150 µ*M*, the rightmost yellow curve was observed. At 0.068 µ*M*, the leftmost blue curve was observed. (*b*) Binding-affinity (*K*_d_) estimation at 44°C, based on the coupling between the unfolding and binding equilibriums. (*c*) Normalized signal versus time. The ‘cold’ (−1 to 0 s) and ‘hot’ (0 to 2 s) regions are depicted by light blue and pink rectangles, respectively. (*d*) Fitting of the *F*_norm_ versus ligand concentration curve. The experimental data points are represented by blue dots and the fitting by an orange line.

To measure the binding affinity between this compound and PknG (a construct comprising the kinase and rubredoxin domains), we analyzed thermal unfolding curves using the isothermal approach. We co-incubated PknG at 15 µ*M* with AX2017 at different concentrations ranging from 150 to 0.068 µ*M* and monitored the change in fluorescence using a temperature ramp from 20 to 90°C (Fig. 6 ▸ *a*). Thermal de­naturation curves for this system show two unfolding transitions: one around 45°C and another less dominant transition at higher temperatures. The first transition is sensitive to addition of the ligand, with the melting temperature moving from 42 to 50°C. We selected a temperature window between 30 and 56°C for isothermal analysis since this region is dominated by the first transition. Each thermal curve (from 30 to 56°C) was individually fitted using the ‘local option’ as described in *FoldAffinity*, and subsequently the equilibrium dissociation constant *K*_d_ was estimated at 44°C using a 1:1 binding model (Fig. 6 ▸ *a*). The temperature for the isothermal analysis needs to be chosen close to the *T*_m_, otherwise the approximation for Δ*H*, which is the basis of the isothermal analysis, is no longer valid. We chose 44°C because it showed the largest amplitude on the unfolded fraction (from almost 1 at the lowest ligand concentration to around 0.2 at the highest ligand concentration; Fig. 6 ▸ *b*), thus allowing a better estimation of *K*_d_. The estimated *K*_d_ was 1.3 µ*M* (Fig. 6 ▸ *b*), which is in good agreement with previous measurements.

Additionally, to measure the binding affinity we performed an MST experiment using PknG labelled with RED-MALEIMIDE 2nd Generation (Cysteine Reactive). In brief, PknG at 25 n*M* was co-incubated with AX2017 at different concentrations ranging from 150 to 0.0046 µ*M* and the fluorescence signal was recorded for around 20 s after laser-induced heating (Fig. 6 ▸ *c*). The estimated equilibrium dissociation constant, also congruent with previous measurements, was 1 µ*M* (Walburger *et al.*, 2004 ▸; Scherr *et al.*, 2007 ▸). It has been proposed that the analysis is more robust when using the initial part of the thermophoresis curve, *i.e.* 0.5–1.5 s, as the ‘hot’ region (López-Méndez, Baron *et al.*, 2021 ▸; López-Méndez, Uebel *et al.*, 2021 ▸). In our case, we obtained an estimation with low error by using the interval 0–2 s as the ‘hot’ region. Similar *K*_d_ values were obtained when selecting different intervals (Supplementary Table S1).

## 3. Conclusions   

Structural determination depends on finding optimal experimental conditions for crystallization and electron-microscopy studies, with protein stability being one of the key factors in success (Deller *et al.*, 2016 ▸). We therefore expect that the online tool *MoltenProt* will help researchers to analyze their DSF (nanoDSF, ThermoFluor) data and to obtain thermodynamic parameters to reliably rank the screened experimental conditions. The addition of an ATP analog (ATPγS) increased the melting temperature of PknG stored in the purification buffer by approximately 1–2°C. In the pH screening, PknG (kinase and rubredoxin domains) was shown to be stable at pH values higher than 6.5.

Additionally, with *FoldAffinity*, estimating binding affinities from nanoDSF data by the isothermal approach requires around 28 capillaries of 10 µl each (two technical replicates for each concentration), totalling 280 µl, with a protein concentration of 0.5–1 mg ml^−1^. The combination of low sample consumption and easy data analysis turns DSF into a useful technique not only for ligand screening but also to quantify biomolecular interactions. In this work, we have shown how to apply isothermal analysis as implemented in *FoldAffinity* to obtain the equilibrium dissociation constant between PknG and the ATP-competitive ligand AX2017. To our knowledge, *FoldAffinity* is the only available online tool to perform isothermal analysis from DSF data.

Finally, *ThermoAffinity* is the first free online tool that allows the quantification of binding affinities from MST experiments. For the PknG–AX2017 interaction we estimated an equilibrium dissociation constant at 25°C which is close to the value obtained from the isothermal analysis of nanoDSF data at 44°C (1.0 versus 1.3 µ*M*). As for the determination with *FoldAffinity*, MST experiments require even less protein than nanoDSF (10 µl per capillary at a final concentration of 500 n*M* for 16 capillaries), but most of the time requires labelling of the sample to obtain good-quality data. Moreover, *ThermoAffinity* has implemented more complex binding models than 1:1 binding, such as two-site models with identical or different equilibrium dissociation constants, and has the flexibility to also analyze fluorescence quenching or enhancement experiments.

In summary, we have presented three virtual tools for the understanding of biophysical data that are beneficial for researchers as they can be accessed online in a centralized fashion, they do not require a programming background and they allow user-friendly, high-quality and reproducible data analysis.

## 4. Methods   

### 4.1. Experimental methods   

#### 4.1.1. Protein expression and purification   

The construct of PknG (kinase and rubredoxin domains) was overexpressed in *Escherichia coli* BL21(DE3) cells. The transformed cells were grown overnight in 10 ml LB medium supplemented with kanamycin (30 µg µl^−1^) at 37°C. The next day, the cells were pelleted by centrifugation at 3500*g* for 10 min and used to inoculate 1 l M9 medium supplemented with 50 µg l^−1^ kanamycin. When the OD_600_ reached ∼0.8–1, protein expression was induced by adding 0.25 m*M* IPTG. Also, to favour iron binding by the rubredoxin domain, we added 100 µ*M* FeCl_3_. The cells were then grown for a further 22 h at 15°C, pelleted by centrifugation at 3500*g* for 10 min, resuspended in 50 m*M* Tris–HCl, 250 m*M* NaCl, 5% glycerol pH 8 and lysed by high pressure in a French press. After clarification by centrifugation at 45 000*g* for 40 min at 10°C, ascorbic acid was added to the supernatant to a final concentration of 1 m*M* to reduce the iron in the rubredoxin domain. The supernatant was then passed through a nickel column and the His-tagged protein was eluted with imidazole. Fractions containing the protein were further purified and the buffer was exchanged to 50 m*M* Tris–HCl, 250 m*M* NaCl, 5% glycerol pH 8 using a HiLoad 16/600 Superdex 200 pg molecular-exclusion column. Fractions with protein were subjected to SDS–PAGE to check for purity, pooled, concentrated to 4 mg ml^−1^ and flash-frozen.

#### 4.1.2. nanoDSF studies   

The change in fluorescence (at 330 and 350 nm) was monitored using a temperature ramp from 20 to 90°C with a Nanotemper Prometheus NT.48 fluorimeter (Nanotemper) controlled by *PR.ThermControl* (version 2.1.2; rate of 1°C min^−1^). Before each experiment, the excitation power was adjusted to achieve fluorescence readings above 2000 RFU. For all measurements, Prometheus NT.48 series nanoDSF-grade standard capillaries (Nanotemper, Munich, Germany) were used.

#### 4.1.3. pH screening (nanoDSF)   

To acquire the melting curves of PknG at different pH values, we used wells E3 to E12 from the RUBIC Buffer Screen (Boivin *et al.*, 2013 ▸; Newman, 2004 ▸). Briefly, SPG buffer (2:7:7 succinic acid/sodium phosphate monobasic monohydrate/glycine) at 100 m*M* was used to explore stability from pH 5.0 to pH 10.0.

#### 4.1.4. Ligand screening (nanoDSF)   

AX2017 was purchased from Sigma–Aldrich. The powder was dissolved in 100% DMSO at a concentration of 100 m*M*. In all of the experiments involving AX2017, the final concentration of DMSO was kept constant at 1%. ATPγS was purchased from Sigma–Aldrich and was solubilized in 50 m*M* Tris–HCl, 250 m*M* NaCl, 5% glycerol at a final concentration of 50 m*M*. 1 *M* MgCl_2_ was prepared in water.

We tested ten different experimental conditions. ‘Control’ refers to the protein stored in the purification buffer. ‘Control + DMSO’, ‘ATPγS’, ‘Mg’, ‘ATPγS + Mg’ and ‘AX2017’ represent the condition ‘Control’ with the addition of 1%(*v*/*v*) DMSO, of ATPγS at 100 µ*M*, of MgCl_2_ at 2 m*M*, of ATPγS at 200 µ*M* and MgCl_2_ at 2 m*M*, and of AX2017 at 40 µ*M*, respectively. ‘CC’ refers to mixing, in a 1:1 ratio, the protein stored in the purification buffer with an experimental condition similar to that used by Lisa *et al.* (2015 ▸) to obtain the crystal structure with PDB code 4y12 [0.2 *M* CaCl_2_, 0.1 *M* Tris pH 8.5, 20%(*w*/*v*) PEG 4000]. ‘CC + DMSO’, ‘CC + AX2017’ and ‘CC + ATPγS’ represent the condition ‘CC’ with the addition of 1%(*v*/*v*) DMSO, of AX2017 at 40 µ*M* and of ATPγS at 100 µ*M*, respectively.

#### 4.1.5. PknG–AX2017 binding affinity (nanoDSF)   

PknG was incubated at 15 µ*M* with AX2017 at concentrations ranging from 150 to 0.068 µ*M* (16 points, 1.5-fold dilution factor and two technical replicates).

#### 4.1.6. PknG–AX2017 binding affinity (labelled MST)   

PknG was labelled using RED-MALEIMIDE 2nd Generation (Cysteine Reactive) (Nanotemper) following the manufacturer’s protocol. The labelling efficiency was 0.52 as determined by measuring the absorbance at 280 and 650 nm.

The labelled MST experiments were performed with a Nanotemper Monolith NT.115 using the *MO.Control* version 1.6 acquisition software and Monolith NT.115 Premium capillaries (MO-K025). The MST power was set to low and the excitation power to 40%, the excitation type was Nano-Red and the temperature was kept constant at 22°C. The protein concentration was 25 n*M*. 16 different ligand concentrations (serial dilution with a factor of 2) in the range between 150 and 0.0046 µ*M* were used for measurements.

### 4.2. Computational methods   

#### 4.2.1. PknG stability estimation with *MoltenProt*   

To estimate the melting temperature at different pH values, each thermal curve was restricted to the 20–56°C range, normalized with the min–max scaling option (*MoltenProt* user documentation, Section 1.2.a) and fitted with the equilibrium two-state unfolding model (*MoltenProt* user documentation, Section 2.1). The same protocol was applied to analyze the stability with different ligands, except for the temperature range, which was fixed to the range 25–60°C.

#### 4.2.2. PknG–AX2017 binding-affinity estimation using *FoldAffinity*   

Each thermal curve was filtered by using lower and upper limits of 30 and 56°C and then individually fitted using the local option as described in *FoldAffinity* with Δ*C*_p_ = 0, *i.e.* assuming that the heat capacity change of unfolding at constant pressure is zero, and subsequently the equilibrium dissociation constant *K*_d_ was estimated at 44°C using the 1:1 binding model.

#### 4.2.3. PknG–AX2017 binding-affinity estimation using *ThermoAffinity*   

The regions for *F*_norm_ determination were −1 to 0 s for ‘cold’ and 0 to 2 s for ‘hot’. The signal was normalized as explained in the documentation. To determine the equilibrium dissociation constant, the 1:1 binding model was used.

## 5. Figures   

The plots shown in Figs. 2 ▸, 3 ▸, 4 ▸, 5 ▸ and 6 ▸ were directly exported from *MoltenProt*, *FoldAffinity* and *ThermoAffinity*. Figs. 2 ▸, 3 ▸ and 4 ▸ were finalized with *Adobe Illustrator* (Adobe). Figs. 5 ▸ and 6 ▸ were finalized with *Inkscape* (https://inkscape.org/).

## Supplementary Material

Supplementary information. DOI: 10.1107/S2059798321008998/qt5002sup1.pdf
