# Supplementary material for: eSPC: an online data-analysis platform for molecular biophysics
Source: Acta Crystallogr D Struct Biol. 2021 Sep 24;77(Pt 10):1241–50. doi: 10.1107/S2059798321008998 (PMC8489228; doi:10.1107/S2059798321008998)
Supplement: Supplementary file 1 [file d-77-01241-sup1.pdf]

STRUCTURAL BIOLOGY

Volume 77 (2021)

Supporting information for article:

eSPC: an online data-analysis platform for molecular biophysics

Osvaldo Burastero, Stephan Niebling, Lucas A. Defelipe, Christian Günther, Angelica Struve and Maria M. Garcia Alai

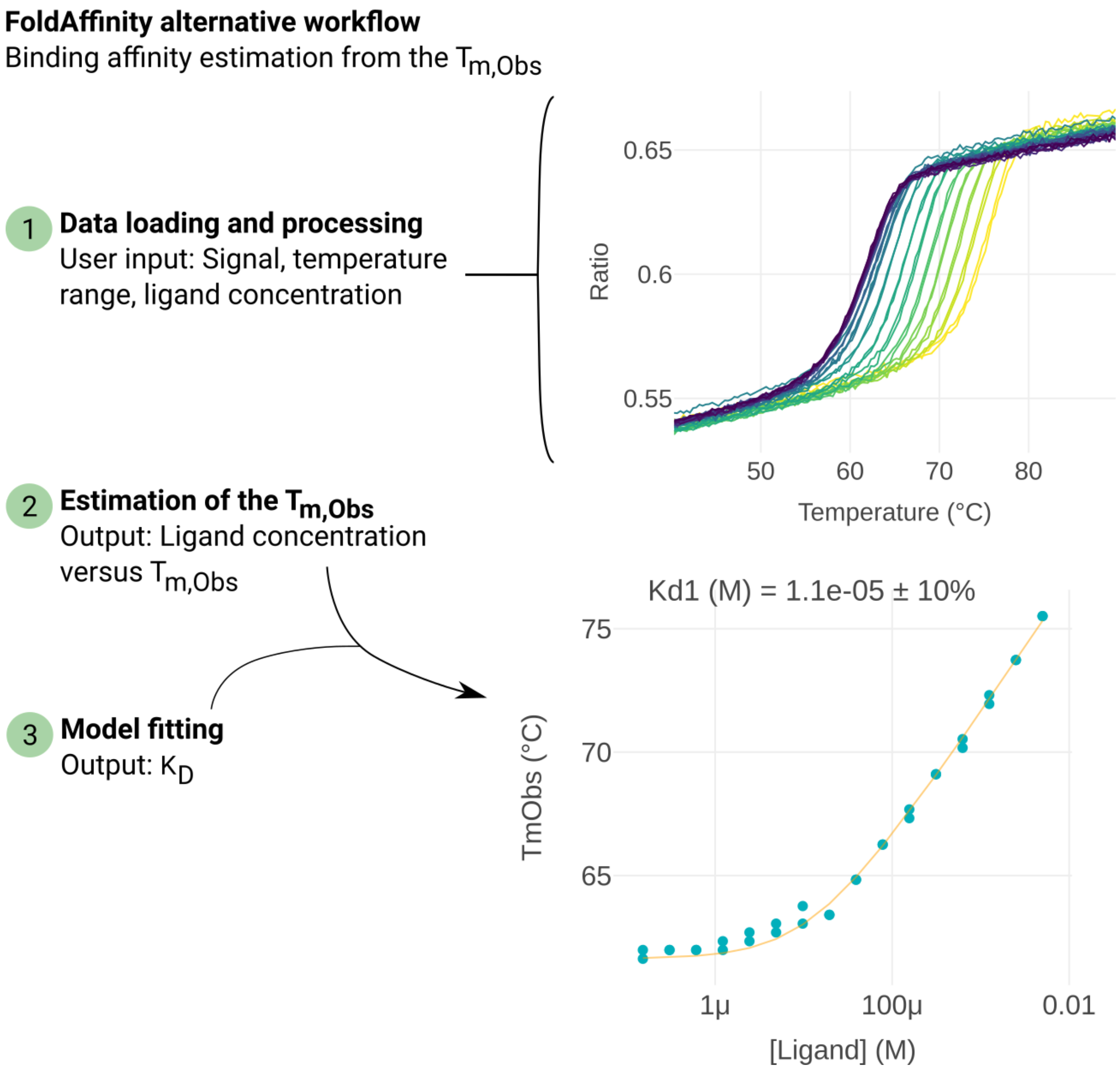

Figure S1. Alternative approach to estimate protein-ligand binding affinities from fluorescence-based melting curves. In the first step, the data is loaded and preprocessed. The preprocessing includes selecting the temperature range, smoothening the data and adding information about the ligand concentration of each capillary/well. The signal versus temperature plot will be color coded using a base-10 log scale and the viridis palette. Second, the observed melting temperature ($T_{m,Obs}$) is obtained from each melting curve using the maximum (or minimum) of the first derivative. Third, the equilibrium dissociation constant K_D is estimated from the ligand concentration versus melting temperature curve. In this particular example, the fitted K_D was 11 µM, which is in close agreement with the K_D derived from the isothermal approach (9.1 µM at 64°C, Figure 3 of the Manuscript).

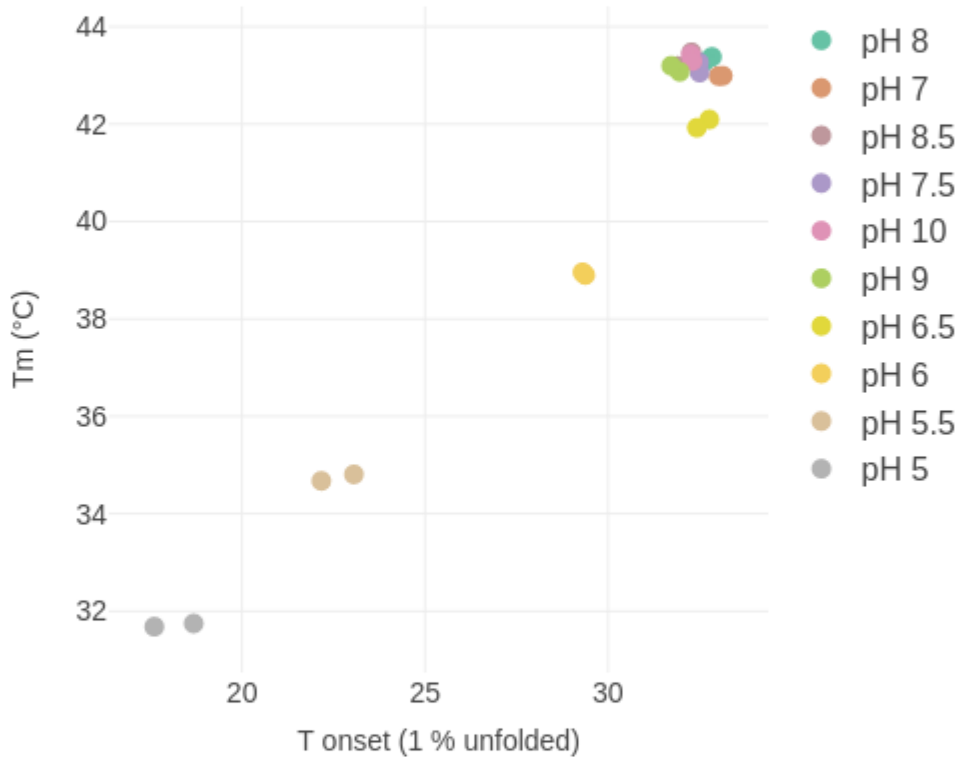

Figure S2. PknG stability starts decreasing at pH 6.5. Melting temperature T_m versus onset temperature T_{onset} at different pHs. T_{onset} is calculated as the temperature where 1 % of the protein is unfolded (using the estimated enthalpy of unfolding ΔH_m and melting temperature T_m).

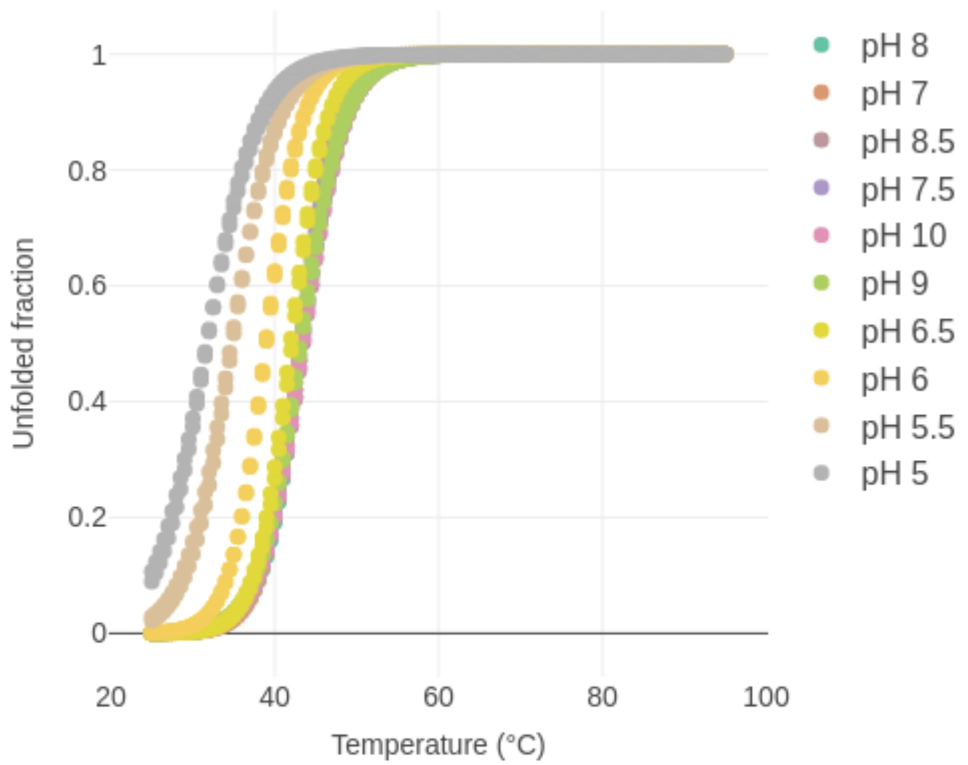

Figure S3. PknG unfolds at lower temperatures with lower pHs. Calculated fraction unfolded (from the estimated enthalpy of unfolding ΔH_m and melting temperature T_m) versus temperature at different pHs. The first six leftmost curves correspond to pH 5, 5, 5.5, 5.5, 6 and 6.

Kd estimation (µM)	Rel. fitting error (%)	95 % CI - Lower (µM)	95 % CI - Upper (µM)	Hot region interval (s)
0.8	28	0.32	1.28	[0-1]
1.05	22	0.55	1.55	[2-3]
1.02	18	0.63	1.41	[4-5]
0.92	20	0.53	1.32	[6-7]
0.76	21	0.41	1.11	[8-9]
0.71	25	0.33	1.08	[10-11]
0.57	34	0.15	0.99	[12-13]

Table S1. Determination of K_d for PknG-AX2017 using different selections for the “Hot region”. Rel. fitting error (%) is calculated as the quotient between the std. error and the estimated value of the fitted parameter. 95 % CI - lower and 95 % CI - upper represent respectively the lower and upper bounds of the 95 % confidence interval.

Appendix - Models implemented in the eSPC platform and their associated equations

MoltenProt

Equilibrium two-state[1,2]

This thermodynamic-based model presupposes that the protein only exists in the native (folded) or unfolded state and that there is an equilibrium between these two states given by the unfolding reaction N ⇆ U.The fluorescence signal $F(T)$ is described by the equation

$$F(T) = (k_n T + b_n + (k_u T + b_u) * e^{\frac{\Delta Hm}{R}(\frac{1}{T_m} - \frac{1}{T})})(1 + e^{\frac{\Delta Hm}{R}(\frac{1}{T_m} - \frac{1}{T})})^{-1} \quad (1)$$

where k_n, b_n are the slope and intercept of the pre-transition baseline (native), k_u and b_u are the slope and intercept of the post-transition (unfolded) baseline, R is the universal gas constant, ΔH_m is the enthalpy of unfolding at the melting temperature T_m. This model assumes that the heat capacity ΔCp of unfolding equals zero. If the user has knowledge about these value, it can be used to correct later the calculated Gibbs energy of unfolding at the standard temperature ($25°C$, 298.15 K) by using

$$\Delta G_{298.15}(\Delta H_m, T_m, C_p) = \Delta H_m * (1 - \frac{298.15\ K}{T_m}) - C_p * (dC_{p,Component}) \quad (2)$$

where

$$dC_{p,Component}(T_m) = T_m - 2918.15\ K\ +\ 298.15\ K\ *\ ln(\frac{298.15\ K}{T_m}) \quad (3)$$

Empirical two-state[3]

[1] Santoro, M. M., & Bolen, D. W. (1988). Unfolding free energy changes determined by the linear extrapolation method. 1. Unfolding of phenylmethanesulfonyl. alpha.-chymotrypsin using different denaturants. *Biochemistry*, *27*(21), 8063-8068.

[2] Bedouelle, H. (2016). Principles and equations for measuring and interpreting protein stability: From monomer to tetramer. *Biochimie*, *121*, 29-37.

[3] Kotov, V., Bartels, K., Veith, K., Josts, I., Subhramanyam, U. K. T., Günther, C., ... & Garcia-Alai, M. M. (2019). High-throughput stability screening for detergent-solubilized membrane proteins. *Scientific reports*, *9*(1), 1-19.

This model is similar to the Equilibrium two-state, but instead of enthalpy of unfolding, it uses the descriptive parameter T_{onset} to describe the steepness of the fluorescence curve. The signal is described by the equation:

$$F(T) = (k_n T + b_n + (k_u T + b_u) * A1(1 + A1)^{-1} \quad (4)$$

where

$$A1 = exp(\frac{(T - T_m) * ln(0.01 / 0.99)}{T_{Onset} - Tm}) \quad (5)$$

Equilibrium three-state[4]

This model adds the presence of one short-lived protein state: native (N), intermediate (I) and unfolded (U). The signal is described by the equation:

$$F(T) = (k_n T + b_n + k_1 A1 + (k_u T + b_u) * A1 * A2)(1 + A1 * A2)^{-1} \quad (6)$$

where

$$A1 = exp(\frac{\Delta H_{m1}}{R}(\frac{1}{T_1} - \frac{1}{T})) \quad (7)$$

and,

$$A2 = exp(\frac{\Delta H_{m2}}{R}(\frac{1}{T_2} - \frac{1}{T})) \quad (8)$$

where k_1 is the signal slope for the short-lived I state, ΔH_{m1} and ΔH_{m2} are the enthalpy of unfolding at melting temperatures T1 (N ⇆ I) and T2 (I ⇆ U).

Empirical three-state[5]

This model is similar to the Equilibrium three-state, but instead of enthalpy of unfolding, it uses the descriptive parameters T_{onset1} and

[4] Mazurenko, S., Kunka, A., Beerens, K., Johnson, C. M., Damborsky, J., & Prokop, Z. (2017). Exploration of protein unfolding by modelling calorimetry data from reheating. *Scientific reports*, *7*(1), 1-14.

[5] Kotov, V., Bartels, K., Veith, K., Josts, I., Subhramanyam, U. K. T., Günther, C., ... & Garcia-Alai, M. M. (2019). High-throughput stability screening for detergent-solubilized membrane proteins. *Scientific reports*, *9*(1), 1-19.

T_{onset2} to describe the steepness of the fluorescence curve. The signal is described by the equation:

$$F(T) = (k_n T + b_n + k_1 A1 + (k_u T + b_u)A1 * A2)(1 + A1 * A2)^{-1} \qquad (9)$$

where

$$A1 = exp(\frac{T - T_1}{T_{Onset1} - T_1} ln(0.01/0.99)) \qquad (10)$$

and,

$$A2 = exp(\frac{T - T_2}{T_{onset2} - T_2} ln(0.01/0.99)) \qquad (11)$$

Irreversible two-state[6],[7]

This model assumes that the protein only exists in the native (N) and unfolded (U) state and that the unfolding reaction is irreversible. The signal is described by the equation:

$$F(T) = k_u T + b_u + (k_n T + b_n) * x_n(T) \qquad (12)$$

where $x_n(T)$ is the fraction of natively folded molecules as a function of temperature and can be obtained via numerical integration:

$$x_n(T) = \int_{Tmin}^{Tmax} \frac{-1}{v} * exp(\frac{-E_a}{R}(\frac{1}{T} - \frac{1}{T_f}) * x_n) \qquad (13)$$

where $Tmax$ and $Tmin$ are the start and end temperatures of the measurement, v is the scan rate in degrees/minutes, E_a is the activation energy of unfolding, T_f is the temperature where the reaction rate constant of unfolding equals 1. For simplicity, x_n is assumed to be 1 at $Tmin$.

[6] Mazurenko, S., Kunka, A., Beerens, K., Johnson, C. M., Damborsky, J., & Prokop, Z. (2017). Exploration of protein unfolding by modelling calorimetry data from reheating. *Scientific reports*, *7*(1), 1-14.

[7] Sanchez-Ruiz, J. M. (1992). Theoretical analysis of Lumry-Eyring models in differential scanning calorimetry. *Biophysical journal*, *61*(4), 921-935.

FoldAffinity

Step 1. Fitting of the fluorescence signal

Each fluorescence versus temperature curve is fitted with a two-state folding model where the signal is the sum of the fluorescence of the folded and unfolded states.

$$F(T) = F_{obs}(IF + T * SF) + U(IU + T * SU) \qquad (14)$$

where F_{obs} and U are respectively the observed folded and unfolded fractions, IF and IU are the intercept of the folded and unfolded fractions, IU and SU are the slope of the folded and unfolded fractions, and

$$F_{obs}(K_{u,obs}) = 1 / (1 + K_{u,obs}) \qquad (15)$$
$$U(K_{u,obs}) = K_{u,obs} / (1 + K_{u,obs}) \qquad (16)$$

with

$$K_{u,obs}(T) = e^{(-\Delta G_{obs} / RT)} = \frac{U}{F+FL} \qquad (17)$$

$$\Delta G_{obs}(T) = \Delta H_{obs} * (1 - \frac{T}{T_{m,obs}+273.15}) + \\ - C_p * (T_{m,obs} + 273.15 - T + T * log(\frac{T}{T_{m,obs}+273.15})) \qquad (18)$$

where R is the gas constant, $\Delta G_{obs}(T)$ is the free energy of unfolding, U is the equilibrium concentration of the unfolded species, F is the equilibrium concentration of the folded unbound species, FL is the equilibrium concentration of the folded bound species, Tm_{obs} is the observed melting temperature, ΔH_{obs} is the enthalpy of unfolding, and C_p is the heat capacity at a constant temperature.

Equation 18 is thermodynamically correct only when there is no ligand involved ($K_{u,obs} = K_u = U / F$). When there is ligand present, $Ku_{obs} = U / (F + FL)$. In spite of this, Bai *et al.* and Niebling *et al.* have

proven that this equation allows later a correct estimation of the equilibrium dissociation constant.[8,9]

Step 2. Fitting of the unfolded fraction versus ligand concentration curve

One binding site

At a fixed temperature, if we assume that the ligand can only bound the folded state, a one binding site system can be described with the following reactions.

$$U + L \Leftrightarrow F + L \Leftrightarrow FL \quad (19)$$

where U, F, FL and L are respectively the unfolded state, folded state, bound folded state and ligand. The associated equations and principle of mass conservation are

$$K_u = U / F \quad (20)$$

$$K_d = (F * L) / FL \quad (21)$$

$$P_0 = U + F + FL \quad (22)$$

$$L_0 = L + FL \quad (23)$$

where K_u and K_d are respectively the unfolding and the equilibrium dissociation constant, P_0 and L_0 are respectively the total protein and total ligand concentration.

If we knew K_u and K_d, FL could be obtained by solving

$$0 = FL^2 + pFL + q \quad (24)$$

$$p = P_0 / (K_u + 1) + L_0 + K_d \quad (25)$$

[8] Bai, N., Roder, H., Dickson, A., & Karanicolas, J. (2019). Isothermal analysis of ThermoFluor data can readily provide quantitative binding affinities. *Scientific reports*, *9*(1), 1-15.

[9] Niebling, S., Burastero, O., Bürgi, J., Günther, C., Defelipe, L. A., Sander, S., ... & García-Alai, M. (2021). FoldAffinity: binding affinities from nDSF experiments. *Scientific reports*, *11*(1), 1-17.

$$q = P_0 L_0 / (K_u + 1) \quad (26)$$

And then U, and F could be calculated to determine the unfolded fraction ($U / (F + FL)$). Therefore, at a fixed temperature, the unfolded fraction versus ligand concentration curve allows to estimate K_u and K_d.

Two binding sites (microscopic constants)

The system is described by the reactions

$$U + 2L \Leftrightarrow F + 2L \quad (27)$$

$$F + 2L \Leftrightarrow FL + L \quad (28)$$

$$F + 2L \Leftrightarrow LF + L \quad (29)$$

$$FL + L \Leftrightarrow LFL \quad (30)$$

$$LF + L \Leftrightarrow LFL \quad (31)$$

where F is the free folded protein, FL and LF are the two possible protein-ligand complexes and LFL is the protein with two ligands. F, FL, LF and LFL depend on the K_ds, total ligand concentration L_0 and free ligand concentration L_{free} in the following way

$$F = K_{d,1} * K_{d,2} * (L_0 - L_{free}) / (K_{d,1} + K_{d,2} + 2L_{free}) / L_{free} \quad (32)$$

$$FL = L_{free} * F / K_{d,2} \quad (33)$$

$$FL = L_{free} * F / K_{d,1} \quad (34)$$

$$LFL = L_{free} * LF / K_{d,2} \quad (35)$$

We can obtain the value of L_{free} by solving

$$X^3 + pX^2 + qX + r = 0 \quad (36)$$

where

$$p = K_{d,1} + K_{d,2} + (2 * P_0 - L_0) \quad (37)$$

$$q = (P_0 - L_0) * (K_{d,1} + K_{d,2}) + K_{d,1} * K_{d,2} * (1 + K_u) \quad (38)$$

$$r = - L_0 * K_{d,1} * K_{d,2} * (1 + K_u) \quad (39)$$

For simplicity, for now we only provide the option to fit this model using $K_{d,1} = K_{d,2}$.

Alternative model - Binding affinity from the observed melting temperatures[10]

If we suppose that the enthalpy (ΔH) and entropy (ΔS) of unfolding do not change significantly in the vicinity of the melting temperature T_m of the protein, and that given that for all ligand concentrations at the observed melting temperature we have (for a one binding site system):

$$\Delta G(T_{m,Obs}) = \Delta H - T_{m,Obs}\Delta S + RT_{m,Obs} * ln(1 + \frac{L_{free}}{K_d}) \qquad (40)$$

and for a two binding sites system,

$$\Delta G(T_{m,Obs}) = \Delta H - T_{m,Obs}\Delta S + RT_{m,Obs} * ln(1 + L_{free} * \frac{L_{free}+K_{d,1}+K_{d,2}}{K_{d,1}*K_{d,2}}) \quad (41)$$

and that at the melting temperature of the protein (without ligand)

$$\Delta H = T_m \Delta S \quad (42)$$

If we approximate the free ligand concentration using the total ligand concentration we can fit the observed melting temperatures by using

$$T_{m,Obs} = T_m(1 - \frac{RT_m ln(1+L_{free} \,/\, K_d)}{\Delta H}\ )^{-1} \qquad (43)$$

if we have one binding site, or

$$T_{m,Obs} = T_m(1 - \Delta H^{-1}(RT_m ln(1 + L_{free} * \frac{L_{free}+K_{d,1}+K_{d,2}}{K_{d,1}*K_{d,2}})))^{-1} \quad (44)$$

in case of two binding sites.

[10] Schellman, J. A. (1975). Macromolecular binding. *Biopolymers: Original Research on Biomolecules*, *14*(5), 999-1018.

ThermoAffinity

The signal is fitted using a simple model where the contribution of the complex and the unbound protein is given by the following equation:

$$Signal(K_d, L_0, P_0) = RF1 * P(K_d, L_0, P_0) + RF2 * PL(K_d, L_0, P_0) \quad (45)$$

where P and PL are respectively the unbound free protein and the bound protein. P_0 and L_0 are respectively the total protein and ligand concentration and K_D is the equilibrium dissociation constant linked to the chemical equilibrium

$$P + L \leftrightarrow PL, \ K_D = (P * L) / PL \quad (46)$$

and

$RF1$ and $RF2$ are parameters that represent the signal per unit of concentration.

Using Equation 46 and the fact that the total ligand and protein concentrations are constant, we can transform the signal to:

$$Signal(K_d, L_0, P_0) = 0.5 * ((K_d + P_0 + L_0) - \sqrt{(K_d + P_0 + L_0)^2 - 4 * P_0 L_0}) * (RF2 - RF1) + RF1 * P_0 \quad (47)$$

Two binding sites

In the case of the binding sites, the signal can be explained by a linear combination of the amount of free unbound protein, right-bound complex (PL), left-bound complex (LP), and double-bound complex (LPL).

$$Signal(L_0, P_0, K_{d,1}, K_{d,2}, cFactor) = RF1 * P + RF2 * PL + RF3 * LP + RF4 * LPL \quad (48)$$

where $RF1$, $RF2$, $RF3$ and $RF4$ are parameters to fit that represent the signal per units of concentration, L_0, P_0, $K_{d,1}$, $K_{d,2}$ and $cFactor$ are respectively the total protein concentration, total ligand concentration, the equilibrium dissociation constant 1, the equilibrium dissociation constant 2, and cooperativity factor. The associated chemical equilibria are

$$P + 2L \leftrightarrow PL + L \quad (49)$$

$$P + 2L \Leftrightarrow LP + L \quad (50)$$
$$PL + L \Leftrightarrow LPL \quad (51)$$
$$LP + L \Leftrightarrow LPL \quad (52)$$

with equations

$$P = K_{d,1} * K_{d,2} * (L_0 - L) /((K_{d,1} + K_{d,2} + 2 * L) * L) \quad (53)$$
$$PL = (L * P / K_{d,2}) \quad (54)$$
$$LP = (L * P / K_{d,1}) \quad (55)$$
$$LPL = \frac{LP*L}{K_{d,2}* cFactor} \quad (56)$$

where L is the free ligand concentration that corresponds to the the only physical root of the equation

$$X^3 + pX^2 + qX + r \quad (57)$$

$$p = [K_{d,1} + K_{d,2} + (2 * P_0 - L_0) / cFactor] * cFactor \quad (58)$$
$$q = [(P_0 - L_0) * (K_{d,1} + K_{d,2}) + K_{d,1} * K_{d,2}] * cFactor \quad (59)$$
$$r = [- L_0 * K_{d,1} * K_{d,2}] * cFactor \quad (60)$$

eSPC, an Online Data Analysis Platform for Molecular Biophysics

MoltenProt 1.0 User Documentation

July 2021

Table of Contents

1. Load input
    1.1. Input file (raw data)
    1.2. Normalization
    1.3. Median filter (smoothing)
    1.4. Savitzky-Golay (SG) window size
    1.5. Melting temperature (Tm) estimation using the first derivative
2. Fitting
    2.1. Model selection
    2.2. Temperature range for baseline estimation
    2.3. Curve fitting
    2.4. Fitting errors
    2.5. Fitting residuals
3. Analyze
    3.1. Baseline separation factor
    3.2. Protein stability score

Overview

MoltenProt has seven panels (Figure 1). Panels 1-3 contain the necessary steps to analyze user data.

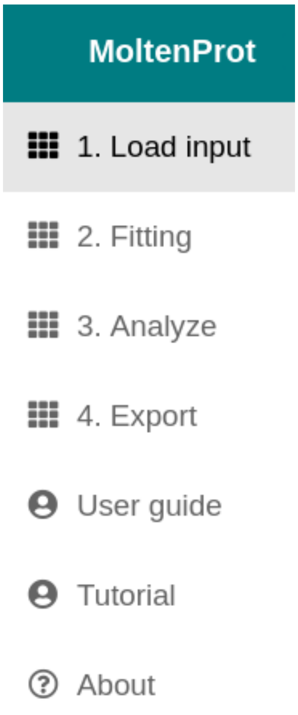

Figure 1. MoltenProt online tool panels.

1. Load input

1.1. Input file (raw data)

MoltenProt accepts as input two kinds of files:

A) The xlsx file (processed) generated by the Nanotemper Prometheus machine that has one sheet called 'Overview' with a column called 'Sample ID' with the names of the samples (Figure 2), and four sheets called 'Ratio', '330nm', '350nm' and 'Scattering'. The first column of the signal sheet ('Ratio', '330nm', '350nm', 'Scattering') should be called 'Time [s]'. The second column should have the temperature data and all subsequent columns store the fluorescence data (Figure 3). The order of the fluorescence columns should match the order of the 'Sample ID' column in the 'Overview' sheet.

B
Sample ID
A1 GuHCl 0.05 M
A2 GuHCl 0.52 M
A3 GuHCl 1 M
A4 GuHCl 1.47 M
A5 GuHCl 1.95 M
A6 GuHCl 2.38 M
A7 GuHCl 2.5 M
A8 GuHCl 2.61 M
A9 GuHCl 2.73 M
A10 GuHCl 2.85 M
A11 GuHCl 2.97 M
A12 GuHCl 3.09 M
B1 GuHCl 3.21 M
B2 GuHCl 3.33 M
B3 GuHCl 3.45 M
B4 GuHCl 3.56 M
B5 GuHCl 3.68 M
B6 GuHCl 4.25 M
B7 GuHCl 4.82 M
B8 GuHCl 5.39 M

Figure 2. Example of the ‘SampleID’ column in the ‘Overview’ sheet required by MoltenProt to load the Nanotemper spreadsheet input file.

	A	B	C	D
1		Capillary	1	2
2		Sample ID	A1	
3	Time [s]	Temperature [°C]	Fluorescence [counts]	Fluorescence [counts]
4	7.0	25.000	0.940	0.934
5	24.3	25.054	0.939	0.935
6	33.3	25.108	0.943	0.933
7	40.6	25.162	0.939	0.936
8	46.8	25.215	0.942	0.933
9	52.5	25.269	0.941	0.933
10	57.4	25.323	0.942	0.934
11	62.1	25.377	0.941	0.934
12	66.7	25.431	0.942	0.934
13	71.0	25.485	0.940	0.933

Figure 3. Example of the ‘Ratio’ sheet required by MoltenProt to load the Nanotemper spreadsheet input file.

B) The xls file generated by the ThermoFluor Assay in a qPCR machine. One sheet called 'RFU' where the first row has the sample positions (header), the first column has the temperature data and all subsequent columns store the fluorescence data (Figure 4).

A	B	C	D	E	F	G	H
	A01	A02	A03	A04	A05	A06	A07
5	64.79	501.82	398.53	61.91	73.26	129.38	38.53
6	63.14	513.32	416.32	63.13	72.41	130.21	40.43
7	61.52	522.98	437.17	64.34	72.21	131.14	42.45
8	59.75	529.89	459.98	64.97	72.52	131.29	42.69
9	57.78	535.95	483.14	65.89	72.30	131.90	43.18
10	55.73	540.72	504.85	67.40	71.83	131.75	42.82
11	54.00	545.15	527.02	68.86	71.27	131.86	42.98
12	52.82	549.80	549.27	70.20	71.55	131.55	42.65
13	52.14	554.45	570.59	70.37	72.86	131.79	42.15
14	51.42	558.53	589.62	70.41	74.39	132.50	41.38

Figure 4. Example of the ‘RFU’ sheet required by MoltenProt to load ThermoFluor data.

1.2. Normalization

There are 3 available options to normalize each fluorescence-based melting curve.

a. Divide by initial value: Divide by the median value of the signal corresponding to the first two degrees of temperature.

b. Max-min normalization: Transform the signal by applying

$$NormalizedSignal(Signal) = \frac{Signal - min(Signal)}{max(Signal) - min(Signal)} \quad \text{Equation 1}$$

c. Area normalization: Divide the signal by the area under the curve (calculated using the trapezoidal rule).

1.3. Median filter (smoothing)

The median filter consists of calculating the median value of a temperature rolling window.

1.4. Savitzky-Golay (SG) window size

This parameter, in degrees Celsius, is used to calculate the number of data points to apply the Savitzky-Golay filter corresponding to a polynomial of degree 4 before computing the first or second derivative as implemented in Scipy (scipy.signal.savgol_filter — SciPy v1.6.1 Reference Guide). For the second derivative, we add 5 degrees to the selected SG temperature window size.

The number of data points is obtained by computing

$$oddDataPoints(SavitzkyGolayWindowSize) = ceil(\frac{SavitzkyGolayWindowSize}{deltaTemperature}) // 2 * 2 + 1$$

Equation 2

where $SavitzkyGolayWindowSize$ is the SG parameter fixed by the user, $ceil(x)$ returns the smallest integer i such that $i >= x$, and $deltaTemperature$ corresponds to the average number of data points in one degree of temperature.

1.5. Melting temperature (T_m) estimation using the first derivative

A non-model approach to estimate the melting temperature involves estimating the maximum or the minimum of the first derivative, depending on the way the signal changes with the temperature. In MoltenProt, the Tm values are estimated as follows. First, the median value of the first derivative in the interval $[min(temperature) + 6; min(temperature) + 11]$ and $[max(temperature) - 11; max(temperature) - 6]$ is calculated. Then, we obtain the mean of those two median values and add it (if it positive), or subtract it (if it is negative), to the first derivative in the interval $[min(temperature) + 6; max(temperature) - 6]$. This is done to shift the derivative baseline. Last, if the absolute value of the minimum (of the derivative) is higher than the absolute value of the maximum, we use the minimum to estimate the Tm. Otherwise, we use the maximum. If many curves are present, we always use the same option.

2. Fitting

2.1. Model selection

The models from the online version of MolteProt are based on the desktop application developed by Kotov et al. (Kotov *et al.*, 2021). All of them assume that the fluorescence signal can be expressed as the sum of the signal from different protein states where the dependence of the signal to the temperature is given by a linear function. The difference in the models lies in establishing which are the possible protein states and how to calculate their concentration (Figure 5).

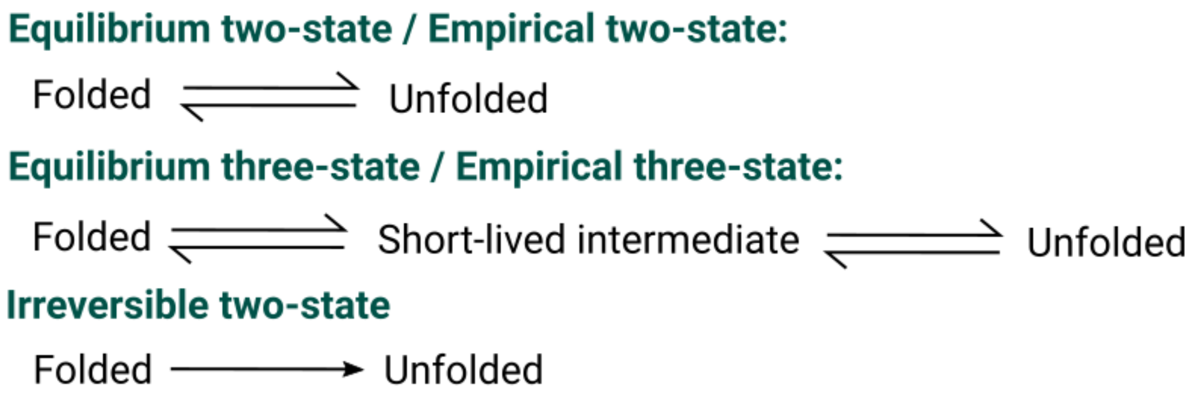

Figure 5. Five unfolding models have been implemented in the online version of MoltenProt. The different protein states are Folded, Unfolded, Short-lived intermediate, and the reaction(s) may be reversible or irreversible.

Equilibrium two-state[1,2]

This thermodynamic-based model presupposes that the protein only exists in the native (folded) or unfolded state and that there is an equilibrium between these two states given by the unfolding reaction N ⇆ U.The fluorescence signal $F(T)$ is described by the equation

$$F(T) = (k_n T + b_n + (k_u T + b_u) * e^{\frac{\Delta H m}{R}(\frac{1}{T_m} - \frac{1}{T})})(1 + e^{\frac{\Delta H m}{R}(\frac{1}{T_m} - \frac{1}{T})})^{-1} \quad (1)$$

where k_n, b_n are the slope and intercept of the pre-transition baseline (native), k_u and b_u are the slope and intercept of the post-transition (unfolded) baseline, R is the universal gas constant, ΔH_m is the enthalpy of unfolding at the melting temperature T_m. This model assumes that the heat capacity ΔC_p of unfolding equals zero. If the user has knowledge about these values, it can be used to correct later the calculated Gibbs energy of unfolding at the standard temperature ($25°C$, 298.15 K) by using

$$\Delta G_{298.15}(\Delta H_m, T_m, C_p) = \Delta H_m * (1 - \frac{298.15\,K}{T_m}) - C_p * (dC_{p,Component}) \quad (2)$$

where

$$dC_{p,Component}(T_m) = T_m - 298.15\,K + 298.15\,K * ln(\frac{298.15\,K}{T_m}) \quad (3)$$

[1] Santoro, M. M., & Bolen, D. W. (1988). Unfolding free energy changes determined by the linear extrapolation method. 1. Unfolding of phenylmethanesulfonyl. alpha.-chymotrypsin using different denaturants. *Biochemistry*, *27*(21), 8063-8068.

[2] Bedouelle, H. (2016). Principles and equations for measuring and interpreting protein stability: From monomer to tetramer. *Biochimie*, *121*, 29-37.

Empirical two-state[3]

This model is similar to the Equilibrium two-state, but instead of enthalpy of unfolding, it uses the descriptive parameter T_{onset} to describe the steepness of the fluorescence curve. The signal is described by the equation:

$$F(T) = (k_n T + b_n + (k_u T + b_u) * A1)(1 + A1)^{-1} \quad (4)$$

where

$$A1 = exp(\frac{(T - T_m) * ln(0.01 / 0.99)}{T_{Onset} - Tm}) \quad (5)$$

Equilibrium three-state[4]

This model adds the presence of one short-lived protein state: native (N), intermediate (I) and unfolded (U). The signal is described by the equation:

$$F(T) = (k_n T + b_n + k_1 A1 + (k_u T + b_u) * A1 * A2)(1 + A1 * A2)^{-1} \qquad (6)$$

where

$$A1 = exp(\frac{\Delta H_{m1}}{R}(\frac{1}{T_1} - \frac{1}{T})) \qquad (7)$$

and,

$$A2 = exp(\frac{\Delta H_{m2}}{R}(\frac{1}{T_2} - \frac{1}{T})) \qquad (8)$$

where k_1 is the signal slope for the short-lived I state, ΔH_{m1} and ΔH_{m2} are the enthalpy of unfolding at melting temperatures T1 (N ⇆ I) and T2 (I ⇆ U).

---

[3] Kotov, V., Bartels, K., Veith, K., Josts, I., Subhramanyam, U. K. T., Günther, C., ... & Garcia-Alai, M. M. (2019). High-throughput stability screening for detergent-solubilized membrane proteins. *Scientific reports*, *9*(1), 1-19.

[4] Mazurenko, S., Kunka, A., Beerens, K., Johnson, C. M., Damborsky, J., & Prokop, Z. (2017). Exploration of protein unfolding by modelling calorimetry data from reheating. *Scientific reports*, *7*(1), 1-14.

Empirical three-state[5]

This model is similar to the Equilibrium three-state, but instead of enthalpy of unfolding, it uses the descriptive parameters T_{onset1} and T_{onset2} to describe the steepness of the fluorescence curve. The signal is described by the equation:

$$F(T) = (k_n T + b_n + k_1 A1 + (k_u T + b_u) A1 * A2)(1 + A1 * A2)^{-1} \qquad (9)$$

where

$$A1 \ = exp(\frac{T - T_1}{T_{Onset1} - T_1} ln(0.01/0.99)) \qquad (10)$$

and,

$$A2 \ = exp(\frac{T - T_2}{T_{onset2} - T_2} ln(0.01/0.99)) \qquad (11)$$

Irreversible two-state[6],[7]

This model assumes that the protein only exists in the native (N) and unfolded (U) state and that the unfolding reaction is irreversible. The signal is described by the equation:

$$F(T) = k_u T + b_u + (k_n T + b_n) * x_n(T) \qquad (12)$$

where $x_n(T)$ is the fraction of natively folded molecules as a function of temperature and can be obtained via numerical integration:

$$x_n(T) = \int_{Tmin}^{Tmax} \frac{-1}{v} * exp(\frac{-E_a}{R}(\frac{1}{T} - \frac{1}{T_f}) * x_n) \qquad (13)$$

where $Tmax$ and $Tmin$ are the start and end temperatures of the measurement, v is the scan rate in degrees/minutes, E_a is the activation

---

[5] Kotov, V., Bartels, K., Veith, K., Josts, I., Subhramanyam, U. K. T., Günther, C., ... & Garcia-Alai, M. M. (2019). High-throughput stability screening for detergent-solubilized membrane proteins. *Scientific reports*, *9*(1), 1-19.

[6] Mazurenko, S., Kunka, A., Beerens, K., Johnson, C. M., Damborsky, J., & Prokop, Z. (2017). Exploration of protein unfolding by modelling calorimetry data from reheating. *Scientific reports*, *7*(1), 1-14.

[7] Sanchez-Ruiz, J. M. (1992). Theoretical analysis of Lumry-Eyring models in differential scanning calorimetry. *Biophysical journal*, *61*(4), 921-935.

energy of unfolding, T_f is the temperature where the reaction rate constant of unfolding equals 1. For simplicity, x_n is assumed to be 1 at $Tmin$.

2.2. Temperature range for baseline estimation

All models require the parameters k_u , b_u, k_n and b_n. The initial values of these parameters are estimated by fitting the equation of a line to the first or last n-degrees (selected by the user).

2.3. Curve fitting

Each curve is fitted individually using the Levenberg Marquardt (damped least-squares) algorithm as implemented in the curve_fit function from the Scipy package. The initial estimates of k_u , b_u, k_n and b_n are used to provide fitting boundaries (± 40 % of the initial values). For the other parameters, the fitting boundaries are described in the following Table.

Parameter	Lower bound	Upper bound
T_m, T_1, T_2	Lowest temperature in data + 6 degrees	Highest temperature in data - 6 degrees
T_onset, T_onset1, T_onset2	Lowest temperature in data + 1 degrees	Highest temperature in data - 11 degrees
dHm, dHm1, dHm2	2.5 kcal/mol	750 kcal/mol
Ki	1e-3	1e3

2.4. Fitting errors

The standard deviation of all fitted parameters is computed using the square root of diagonal values from the fit parameter covariance matrix reported by scipy.curve_fit function. These values are an approximation (underestimation) of the real errors.

2.5. Fitting residuals

The residuals of the fitting are normalized by dividing them by the standard error.

3. Analyze

3.1. Baseline separation factor

This value is useful to compare the height of the unfolding transition for the equilibrium or empirical two-state unfolding models and is calculated as

$$BS(S, k_u, T_m, b_u, k_n, b_n) = 1 - \frac{6*S}{k_u T_m + b_u - k_n * T_m - b_n} \quad (14)$$

where S is the standard error of the estimation.

3.2. Protein stability score

After fitting a model, a protein stability score is provided which can be used to sort the conditions.

Model	Score
Equilibrium two-state	$\Delta G\ of\ unfolding\ at\ 298.15\ K$
Empirical two-state	$distance((T_m; T_{Onset}), (0; 0))$
Equilibrium three-state	$\Delta G\ of\ unfolding\ at\ 298.15\ K$
	$(\Delta G\ of\ reaction\ N \leftrightarrows I + \Delta G\ of\ reaction\ I \leftrightarrows U)$
Empirical two-state	$distance((T_{m1}; T_{Onset1}), (0; 0)) + distance((T_{m2}; T_{Onset2}), (0; 0))$
Irreversible two-state	$-\log(k_{irrev}(298.15\ K))$

References

Kotov, V., Mlynek, G., Vesper, O., Pletzer, M., Wald, J., Teixeira-Duarte, C. M., Celia, H., Garcia-Alai, M., Nussberger, S., Buchanan, S. K., Morais-Cabral, J. H., Loew, C., Djinovic-Carugo, K. & Marlovits, T. C. (2021). *Protein Sci.* **30**, 201–217.

Packages

MoltenProt is possible thanks to:

R language: R Core Team (2020). R: A language and environment for statistical computing. R Foundation for Statistical Computing, Vienna, Austria. URL https://www.R-project.org/.

R package shiny: Winston Chang, Joe Cheng, JJ Allaire, Yihui Xie and Jonathan McPherson (2020). shiny: Web Application Framework for R. R package version 1.4.0.2. https://CRAN.R-project.org/package=shiny

R package viridis: Simon Garnier (2018). viridis: Default Color Maps from 'matplotlib'. R package version 0.5.1. https://CRAN.R-project.org/package=viridis

R package tidyverse: Wickham et al., (2019). Welcome to the tidyverse. Journal of Open Source Software, 4(43), 1686, https://doi.org/10.21105/joss.01686

R package pracma: Hans W. Borchers (2019). pracma: Practical Numerical Math Functions. R package version 2.2.9. https://CRAN.R-project.org/package=pracma

R package shinydashboard: Winston Chang and Barbara Borges Ribeiro (2018). shinydashboard: Create Dashboards with 'Shiny'. R package version 0.7.1. https://CRAN.R-project.org/package=shinydashboard

R package ggplot2: H. Wickham. ggplot2: Elegant Graphics for Data Analysis. Springer-Verlag New York, 2016.

R package xlsx: Adrian Dragulescu and Cole Arendt (2020). xlsx: Read, Write, Format Excel 2007 and Excel 97/2000/XP/2003 Files. R package version 0.6.3. https://CRAN.R-project.org/package=xlsx

R package reshape2: Hadley Wickham (2007). Reshaping Data with the reshape Package. Journal of Statistical Software, 21(12), 1-20. URL http://www.jstatsoft.org/v21/i12/.

R package tippy: John Coene (2018). tippy: Add Tooltips to 'R markdown' Documents or 'Shiny' Apps. R package version 0.0.1. https://CRAN.R-project.org/package=tippy

R package shinyalert: Pretty Popup Messages (Modals) in 'Shiny'. R package version 1.1. https://CRAN.R-project.org/package=shinyalert

R package plotly: C. Sievert. Interactive Web-Based Data Visualization with R, plotly, and shiny. Chapman and Hall/CRC Florida, 2020.

R package tableHTML: Theo Boutaris, Clemens Zauchner and Dana Jomar (2019). tableHTML: A Tool to Create HTML Tables. R package version 2.0.0. https://CRAN.R-project.org/package=tableHTML

R package rhandsontable: Jonathan Owen (2018). rhandsontable: Interface to the 'Handsontable.js' Library. R package version 0.3.7. https://CRAN.R-project.org/package=rhandsontable

R package remotes: Jim Hester, Gábor Csárdi, Hadley Wickham, Winston Chang, Martin Morgan and Dan Tenenbaum (2020). remotes: R Package

Installation from Remote Repositories, Including 'GitHub'. R package version 2.1.1. https://CRAN.R-project.org/package=remotes

R package devtools: Hadley Wickham, Jim Hester and Winston Chang (2020). devtools: Tools to Make Developing R Packages Easier. R package version 2.3.0. https://CRAN.R-project.org/package=devtools

R package shinyjs: Dean Attali (2020). shinyjs: Easily Improve the User Experience of Your Shiny Apps in Seconds. R package version 1.1. https://CRAN.R-project.org/package=shinyjs

R package data.table: Matt Dowle and Arun Srinivasan (2019). data.table: Extension of data.frame. R package version 1.12.8. https://CRAN.R-project.org/package=data.table

R package reticulate: Kevin Ushey, JJ Allaire and Yuan Tang (2020). reticulate: Interface to 'Python'. R package version 1.16. https://CRAN.R-project.org/package=reticulate

R package shinycssloaders: Andras Sali and Dean Attali (2020). shinycssloaders: Add CSS Loading Animations to 'shiny' Outputs. R package version 0.3. https://CRAN.R-project.org/package=shinycssloaders

Baptiste Auguie (2019). egg: Extensions for 'ggplot2': Custom Geom, Custom Themes, Plot Alignment, Labelled Panels, Symmetric Scales, and Fixed Panel Size. R package version 0.4.5. https://CRAN.R-project.org/package=egg

Python3.7 language: Van Rossum, G., & Drake, F. L. (2009). Python 3 Reference Manual. Scotts Valley, CA: CreateSpace.

Python package numpy: Travis E, Oliphant. A guide to NumPy, USA: Trelgol Publishing, (2006). Stéfan van der Walt, S. Chris Colbert, and Gaël Varoquaux. The NumPy Array: A Structure for Efficient Numerical Computation, Computing in Science & Engineering, 13, 22-30 (2011), DOI:10.1109/MCSE.2011.37

Python package pandas: Wes McKinney. Data Structures for Statistical Computing in Python, Proceedings of the 9th Python in Science Conference, 51-56 (2010)

Python package scipy: Pauli Virtanen, Ralf Gommers, Travis E. Oliphant, Matt Haberland, Tyler Reddy, David Cournapeau, Evgeni Burovski, Pearu Peterson, Warren Weckesser, Jonathan Bright, Stéfan J. van der Walt,

Matthew Brett, Joshua Wilson, K. Jarrod Millman, Nikolay Mayorov, Andrew R. J. Nelson, Eric Jones, Robert Kern, Eric Larson, CJ Carey, İlhan Polat, Yu Feng, Eric W. Moore, Jake VanderPlas, Denis Laxalde, Josef Perktold, Robert Cimrman, Ian Henriksen, E.A. Quintero, Charles R Harris, Anne M. Archibald, Antônio H. Ribeiro, Fabian Pedregosa, Paul van Mulbregt, and SciPy 1.0 Contributors. (2020) SciPy 1.0: Fundamental Algorithms for Scientific Computing in Python. Nature Methods, 17(3), 261-272.

Python package xlrd: https://xlrd.readthedocs.io/en/latest/index.html

Python package natsort: https://natsort.readthedocs.io/en/master/

eSPC, an Online Data Analysis Platform for Molecular Biophysics

FoldAffinity 1.0 User Documentation

July 2021

Table of Contents

1. Load input
    1.1. Input file (raw data)
    1.2. Median filter (smoothing)
    1.3. Melting temperature (Tm) estimation using the first derivative
2. Fit fluorescence
    2.1. Model
    2.2. Curve fitting
    2.3. Fitting errors
3. Fit unfolded fraction
    3.1. Models
    3.2. Curve fitting
    3.3. Fitting errors
4. Alternative Tm fitting: Binding affinity from the observed melting temperatures
    4.1 Model
    4.2. Curve fitting
    4.3. Fitting errors

Overview

FoldAffinity has 9 panels (Figure 1). Panels 1-3 contain the necessary steps to analyze user data using the isothermal approach. The Panel Tm fitting can be used to estimate binding affinities directly from the observed melting temperatures. The Simulate data Panel can be used before doing an experiment to analyze the expected change in the signal depending on the binding affinity and the protein and ligand concentrations.

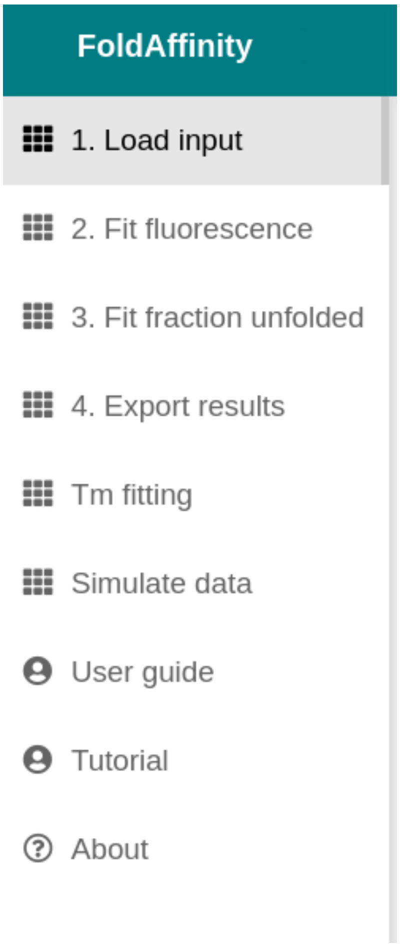

Figure 1. FoldAffinity online tool panels.

1. Load input

1.1. Input file (raw data)

FoldAffinity accepts as input two kind of files:

A) The xlsx file (processed) generated by the Nanotemper Prometheus machine that has one sheet called 'Overview' with a column called 'Sample ID' with the names of the samples (ligand concentration) (Figure 2), and four sheets called 'Ratio', '330nm', '350nm' and 'Scattering'. The first column of the signal sheet ('Ratio', '330nm',

'350nm', 'Scattering') should be called 'Time [s]'. The second column should have the temperature data and all subsequent columns store the fluorescence data (Figure 3). The order of the fluorescence columns should match the order of the 'Sample ID' column in the 'Overview' sheet.

B
Sample ID
A1 GuHCl 0.05 M
A2 GuHCl 0.52 M
A3 GuHCl 1 M
A4 GuHCl 1.47 M
A5 GuHCl 1.95 M
A6 GuHCl 2.38 M
A7 GuHCl 2.5 M
A8 GuHCl 2.61 M
A9 GuHCl 2.73 M
A10 GuHCl 2.85 M
A11 GuHCl 2.97 M
A12 GuHCl 3.09 M
B1 GuHCl 3.21 M
B2 GuHCl 3.33 M
B3 GuHCl 3.45 M
B4 GuHCl 3.56 M
B5 GuHCl 3.68 M
B6 GuHCl 4.25 M
B7 GuHCl 4.82 M
B8 GuHCl 5.39 M

Figure 2. Example of the ‘SampleID’ column in the ‘Overview’ sheet required by FoldAffinity to load the Nanotemper spreadsheet input file.

	A	B	C	D
1		Capillary	1	2
2		Sample ID	A1	
3	Time [s]	Temperature [°C]	Fluorescence [counts]	Fluorescence [counts]
4	7.0	25.000	0.940	0.934
5	24.3	25.054	0.939	0.935
6	33.3	25.108	0.943	0.933
7	40.6	25.162	0.939	0.936
8	46.8	25.215	0.942	0.933
9	52.5	25.269	0.941	0.933
10	57.4	25.323	0.942	0.934
11	62.1	25.377	0.941	0.934
12	66.7	25.431	0.942	0.934
13	71.0	25.485	0.940	0.933

Figure 3. Example of the ‘Ratio’ sheet required by FoldAffinity to load the Nanotemper spreadsheet input file.

B) The xls file generated by the ThermoFluor Assay in a qPCR machine. One sheet called 'RFU' where the first row has the sample positions (header), the first column has the temperature data and all subsequent columns store the fluorescence data (Figure 4).

A	B	C	D	E	F	G	H
	A01	A02	A03	A04	A05	A06	A07
5	64.79	501.82	398.53	61.91	73.26	129.38	38.53
6	63.14	513.32	416.32	63.13	72.41	130.21	40.43
7	61.52	522.98	437.17	64.34	72.21	131.14	42.45
8	59.75	529.89	459.98	64.97	72.52	131.29	42.69
9	57.78	535.95	483.14	65.89	72.30	131.90	43.18
10	55.73	540.72	504.85	67.40	71.83	131.75	42.82
11	54.00	545.15	527.02	68.86	71.27	131.86	42.98
12	52.82	549.80	549.27	70.20	71.55	131.55	42.65
13	52.14	554.45	570.59	70.37	72.86	131.79	42.15
14	51.42	558.53	589.62	70.41	74.39	132.50	41.38

Figure 4. Example of the 'RFU' sheet required by FoldAffinity to load ThermoFluor data.

1.2. Median filter (smoothing)

The median filter consists of calculating the median value of a temperature rolling window.

1.3. Melting temperature (Tm) estimation using the first derivative

A non-model approach to estimate the melting temperature involves estimating the maximum or the minimum of the first derivative, depending on the way the signal changes with the temperature. In FoldAffinity, the Tm values are estimated as follows. First, the median value of the first derivative in the interval $[min(temperature)+6; min(temperature)+11]$ and $[max(temperature)-11; max(temperature)-6]$ is calculated. Then, we obtain the mean of those two median values and add it (if it positive), or subtract it (if it is negative), to the first derivative in the interval $[min(temperature)+6; max(temperature)-6]$. This is done to shift the derivative baseline. Last, if the absolute value of the minimum (of the derivative) is higher than the absolute value of the maximum, we use the minimum to estimate the Tm. Otherwise, we use the maximum. If many curves are present, we always use the same option.

To compute the derivative we use the Savitzky-Golay function as implemented in numpy ([scipy.signal.savgol_filter — SciPy v1.6.1 Reference Guide]) with a polynomial degree 4 and window size of 10 degrees.

2. Fit fluorescence

2.1. Model

Each fluorescence versus temperature curve is fitted with a two-state folding model where the signal is the sum of the fluorescence of the folded and unfolded states.

$$F(T) = F_{obs}(IF + T * SF) + U(IU + T * SU) \quad (1)$$

where F_{obs} and U are respectively the observed folded and unfolded fractions, IF and IU are the intercept of the folded and unfolded fractions, IU and SU are the slope of the folded and unfolded fractions, and

$$F_{obs}(K_{u,obs}) = 1 / (1 + K_{u,obs}) \quad (2)$$
$$U(K_{u,obs}) = K_{u,obs} / (1 + K_{u,obs}) \quad (3)$$

with

$$K_{u,obs}(T) = e^{(-\Delta G_{obs} / RT)} = \frac{U}{F+FL} \quad (4)$$

$$\Delta G_{obs}(T) = \Delta H_{obs} * (1 - \frac{T}{T_{m,obs}+273.15}) + $$
$$- C_p * (T_{m,obs} + 273.15 - T + T * log(\frac{T}{T_{m,obs}+273.15})) \quad (5)$$

where R is the gas constant, $\Delta G_{obs}(T)$ is the free energy of unfolding, U is the equilibrium concentration of the unfolded species, F is the equilibrium concentration of the folded unbound species, FL is the equilibrium concentration of the folded bound species, Tm_{obs} is the observed melting temperature, ΔH_{obs} is the enthalpy of unfolding, and C_p is the heat capacity at a constant temperature.

Equation 18 is thermodynamically correct only when there is no ligand involved ($K_{u,obs} = K_u = U / F$). When there is ligand present, $Ku_{obs} = U / (F + FL)$. In spite of this, Bai *et al.* and Niebling *et al.* have

proven that this equation allows latter a correct estimation of the equilibrium dissociation constant.[1,2]

2.2. Curve fitting

Once the data is loaded in FoldAffinity, the first and last 10 degrees of each fluorescence melting curve is fitted using the equation of a line to obtain initial values of $InterceptFolded$, $SlopeFolded$, $InterceptUnfolded$ and $SlopeUnfolded$ parameters. Then, each curve is fitted individually using the Levenberg Marquardt (damped least-squares) algorithm to estimate ΔH_{obs}, $T_{m,obs}$ and C_p. These values can be used directly or the whole data can be fitted again to force shared values of the slope parameters and / or C_p value.

2.3. Fitting errors

The standard deviation of all fitted parameters is computed using the square root of diagonal values from the fit parameter covariance matrix reported by scipy.curve_fit function. These values are an approximation (underestimation) of the real errors.

3. Fit unfolded fraction

3.1. Models

The models implemented in FoldAffinity are based on the coupling between ligand binding and protein folding and require that the ligand is completely soluble at all the measured concentrations and temperatures.

One binding site

At a fixed temperature, if we assume that the ligand can only bound the folded state, a one binding site system can be described with the following reactions.

$$U + L \Leftrightarrow F + L \Leftrightarrow FL \qquad (6)$$

---

[1] Bai, N., Roder, H., Dickson, A., & Karanicolas, J. (2019). Isothermal analysis of ThermoFluor data can readily provide quantitative binding affinities. *Scientific reports*, *9*(1), 1-15.

[2] Niebling, S., Burastero, O., Bürgi, J., Günther, C., Defelipe, L. A., Sander, S., ... & García-Alai, M. (2021). FoldAffinity: binding affinities from nDSF experiments. *Scientific reports*, *11*(1), 1-17.

where U, F, FL and L are respectively the unfolded state, folded state, bound folded state and ligand. The associated equations and principle of mass conservation are

$$K_u = U / F \quad (7)$$
$$K_d = (F * L) / FL \quad (8)$$
$$P_0 = U + F + FL \quad (9)$$
$$L_0 = L + FL \quad (10)$$

where K_u and K_d are respectively the unfolding and the equilibrium dissociation constant, P_0 and L_0 are respectively the total protein and total ligand concentration.

If we knew K_u and K_d, FL could be obtained by solving

$$0 = FL^2 + pFL + q \quad (11)$$
$$p = P_0 / (K_u + 1) + L_0 + K_d \quad (12)$$
$$q = P_0 L_0 / (K_u + 1) \quad (13)$$

And then U, and F could be calculated to determine the unfolded fraction ($U / (F + FL)$). Therefore, at a fixed temperature, the unfolded fraction versus ligand concentration curve allows to estimate K_u and K_d.

Two binding sites (microscopic constants)

The system is described by the reactions

$$U + 2L \Leftrightarrow F + 2L \quad (14)$$
$$F + 2L \Leftrightarrow FL + L \quad (15)$$
$$F + 2L \Leftrightarrow LF + L \quad (16)$$
$$FL + L \Leftrightarrow LFL \quad (17)$$
$$LF + L \Leftrightarrow LFL \quad (18)$$

where F is the free folded protein, FL and LF are the two possible protein-ligand complexes and LFL is the protein with two ligands. F, FL, LF and LFL depend on the Kds, total ligand concentration L_0 and free ligand concentration L_{free} in the following way

$$F = K_{d,1} * K_{d,2} * (L_0 - L_{free}) / (K_{d,1} + K_{d,2} + 2L_{free}) / L_{free} \quad (19)$$
$$FL = L_{free} * F / K_{d,2} \quad (20)$$

$$FL = L_{free} * F \ / \ K_{d,1} \quad (21)$$
$$LFL = L_{free} * LF \ / \ K_{d,2} \quad (22)$$

We can obtain the value of L_{free} by solving

$$X^3 + pX^2 + qX + r = 0 \quad (23)$$

where

$$p = K_{d,1} + K_{d,2} + (2 * P_0 - L_0) \quad (24)$$
$$q = (P_0 - L_0) * (K_{d,1} + K_{d,2}) + K_{d,1} * K_{d,2} * (1 + K_u) \quad (25)$$
$$r = - L_0 * K_{d,1} * K_{d,2} * (1 + K_u) \quad (26)$$

For simplicity, for now we only provide the option to fit this model using $K_{d,1} = K_{d,2}$.

3.2. Curve fitting

The unfolded fraction versus ligand concentration curve is fitted using the Levenberg Marquardt (damped least-squares) algorithm to estimate K_d (or $K_{d,1}$, $K_{d,2}$) and K_u at the chosen temperature.

3.3. Fitting errors

Explained in section 2.3.

4. Alternative Tm fitting: Binding affinity from the observed melting temperatures

4.1 Model

If we suppose that the enthalpy (ΔH) and entropy (ΔS) of unfolding do not change significantly in the vicinity of the melting temperature T_m of the protein, and that given that for all ligand concentrations at the observed melting temperature we have (for a one binding site system):

$$\Delta G(T_{m,Obs}) = \Delta H - T_{m,Obs}\Delta S + RT_{m,Obs} * ln(1 + \frac{L_{free}}{Kd}) \quad (27)$$

and for a two binding sites system,

$$\Delta G(T_{m,Obs}) = \Delta H - T_{m,Obs}\Delta S + RT_{m,Obs} * ln(1 + L_{free} * \frac{L_{free}+K_{d,1}+K_{d,2}}{K_{d,1}*K_{d,2}}) \quad (28)$$

and that at the melting temperature of the protein (without ligand)

$$\Delta H = T_m \Delta S \quad (29)$$

If we approximate the free ligand concentration using the total ligand concentration we can fit the observed melting temperatures by using

$$T_{m,Obs} = T_m (1 - \frac{RT_m ln(1+L_{free} / K_d)}{\Delta H})^{-1} \quad (30)$$

if we have one binding site, or

$$T_{m,Obs} = T_m (1 - \Delta H^{-1}(RT_m ln(1 + L_{free} * \frac{L_{free}+K_{d,1}+K_{d,2}}{K_{d,1}*K_{d,2}})))^{-1} \quad (31)$$

in case of two binding sites.

4.2. Curve fitting

The observed melting temperature calculated from the first derivative as a function of the total ligand concentration is fitted using the Levenberg Marquardt (damped least-squares) algorithm to estimate ΔH and K_d (or $K_{d,1}$, $K_{d,2}$).

4.3. Fitting errors

Explained in section 2.3.

Packages

FoldAffinity is possible thanks to:

R language: R Core Team (2020). R: A language and environment for statistical computing. R Foundation for Statistical Computing, Vienna, Austria. URL https://www.R-project.org/.

R package shiny: Winston Chang, Joe Cheng, JJ Allaire, Yihui Xie and Jonathan McPherson (2020). shiny: Web Application Framework for R. R package version 1.4.0.2. https://CRAN.R-project.org/package=shiny

R package viridis: Simon Garnier (2018). viridis: Default Color Maps from 'matplotlib'. R package version 0.5.1. https://CRAN.R-project.org/package=viridis

R package tidyverse: Wickham et al., (2019). Welcome to the tidyverse. Journal of Open Source Software, 4(43), 1686, https://doi.org/10.21105/joss.01686

R package pracma: Hans W. Borchers (2019). pracma: Practical Numerical Math Functions. R package version 2.2.9. https://CRAN.R-project.org/package=pracma

R package shinydashboard: Winston Chang and Barbara Borges Ribeiro (2018). shinydashboard: Create Dashboards with 'Shiny'. R package version 0.7.1. https://CRAN.R-project.org/package=shinydashboard

R package ggplot2: H. Wickham. ggplot2: Elegant Graphics for Data Analysis. Springer-Verlag New York, 2016.

R package xlsx: Adrian Dragulescu and Cole Arendt (2020). xlsx: Read, Write, Format Excel 2007 and Excel 97/2000/XP/2003 Files. R package version 0.6.3. https://CRAN.R-project.org/package=xlsx

R package reshape2: Hadley Wickham (2007). Reshaping Data with the reshape Package. Journal of Statistical Software, 21(12), 1-20. URL http://www.jstatsoft.org/v21/i12/.

R package tippy: John Coene (2018). tippy: Add Tooltips to 'R markdown' Documents or 'Shiny' Apps. R package version 0.0.1. https://CRAN.R-project.org/package=tippy

R package shinyalert: Pretty Popup Messages (Modals) in 'Shiny'. R package version 1.1. https://CRAN.R-project.org/package=shinyalert

R package plotly: C. Sievert. Interactive Web-Based Data Visualization with R, plotly, and shiny. Chapman and Hall/CRC Florida, 2020.

R package tableHTML: Theo Boutaris, Clemens Zauchner and Dana Jomar (2019). tableHTML: A Tool to Create HTML Tables. R package version 2.0.0. https://CRAN.R-project.org/package=tableHTML

R package rhandsontable: Jonathan Owen (2018). rhandsontable: Interface to the 'Handsontable.js' Library. R package version 0.3.7. https://CRAN.R-project.org/package=rhandsontable

R package remotes: Jim Hester, Gábor Csárdi, Hadley Wickham, Winston Chang, Martin Morgan and Dan Tenenbaum (2020). remotes: R Package Installation from Remote Repositories, Including 'GitHub'. R package version 2.1.1. https://CRAN.R-project.org/package=remotes

R package devtools: Hadley Wickham, Jim Hester and Winston Chang (2020). devtools: Tools to Make Developing R Packages Easier. R package version 2.3.0. https://CRAN.R-project.org/package=devtools

R package shinyjs: Dean Attali (2020). shinyjs: Easily Improve the User Experience of Your Shiny Apps in Seconds. R package version 1.1. https://CRAN.R-project.org/package=shinyjs

R package data.table: Matt Dowle and Arun Srinivasan (2019). data.table: Extension of data.frame. R package version 1.12.8. https://CRAN.R-project.org/package=data.table

R package reticulate: Kevin Ushey, JJ Allaire and Yuan Tang (2020). reticulate: Interface to 'Python'. R package version 1.16. https://CRAN.R-project.org/package=reticulate

R package shinycssloaders: Andras Sali and Dean Attali (2020). shinycssloaders: Add CSS Loading Animations to 'shiny' Outputs. R package version 0.3. https://CRAN.R-project.org/package=shinycssloaders

Python3.7 language: Van Rossum, G., & Drake, F. L. (2009). Python 3 Reference Manual. Scotts Valley, CA: CreateSpace.

Python package numpy: Travis E, Oliphant. A guide to NumPy, USA: Trelgol Publishing, (2006). Stéfan van der Walt, S. Chris Colbert, and Gaël Varoquaux. The NumPy Array: A Structure for Efficient Numerical Computation, Computing in Science & Engineering, 13, 22-30 (2011), DOI:10.1109/MCSE.2011.37

Python package pandas: Wes McKinney. Data Structures for Statistical Computing in Python, Proceedings of the 9th Python in Science Conference, 51-56 (2010)

Python package scipy: Pauli Virtanen, Ralf Gommers, Travis E. Oliphant, Matt Haberland, Tyler Reddy, David Cournapeau, Evgeni Burovski, Pearu

Peterson, Warren Weckesser, Jonathan Bright, Stéfan J. van der Walt, Matthew Brett, Joshua Wilson, K. Jarrod Millman, Nikolay Mayorov, Andrew R. J. Nelson, Eric Jones, Robert Kern, Eric Larson, CJ Carey, İlhan Polat, Yu Feng, Eric W. Moore, Jake VanderPlas, Denis Laxalde, Josef Perktold, Robert Cimrman, Ian Henriksen, E.A. Quintero, Charles R Harris, Anne M. Archibald, Antônio H. Ribeiro, Fabian Pedregosa, Paul van Mulbregt, and SciPy 1.0 Contributors. (2020) SciPy 1.0: Fundamental Algorithms for Scientific Computing in Python. Nature Methods, 17(3), 261-272.

Python package xlrd: https://xlrd.readthedocs.io/en/latest/index.html

Python package natsort: https://natsort.readthedocs.io/en/master/

eSPC, an Online Data Analysis Platform for Molecular Biophysics

ThermoAffinity 1.0 User Documentation

July 2021

Table of Contents

1. Load input
    1.1. Input file (raw data)
    1.2. Normalization
    1.3. Median filter (smoothing)
    1.4. Hot and cold region selection
2. Fitting
    2.1 Model
    2.2 Initial estimates and boundaries of the parameters
    2.3 Curve fitting
    2.4 Fitting errors

Overview

ThermoAffinity has seven panels (Figure 1). Panels 1-2 contain the necessary steps to analyze user data. The Simulate data Panel can be used before doing an experiment to analyze the expected change in the signal depending on the binding affinity and the protein and ligand concentrations.

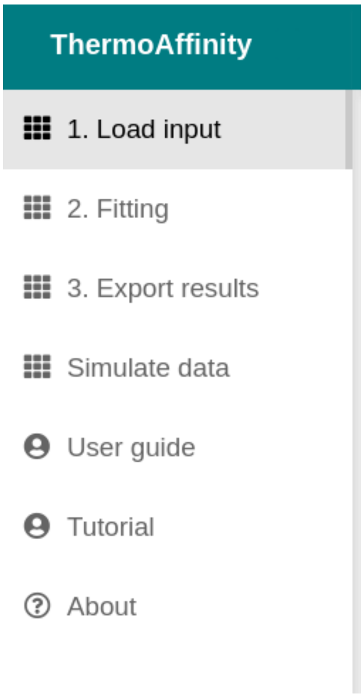

Figure 1. ThermoAffinity online tool panels.

1. Load input

1.1. Input file (raw data)

ThermoAffinity accepts as input the spreadsheet generated by NanoTemper Technologies. This file contains one sheet called 'RawData' where the first column has different cells with the following labels: 'Capillary Position:', 'Ligand:', 'Ligand Concentration:', and 'Time [s]'. Then, the second column stores the associated information: '1', 'ligand description', '5000', and 'Raw Fluorescence [counts]' (Figure 2). The next capillary information is going to be read from columns 4 and 5, then from columns 7 and 8, etc.

Origin of exported data			
Project Title:			
Comment:			
Project File Path:			
Analysis-Set Name:			
Exported from:	MST Traces (Raw Data Inspection)		
Exported on:	2020-09-14 15:25:28.999		
**Sample Information**		**Sample Information**	
Merge-Set Name:		Merge-Set Name:	
Run Name:		Run Name:	
ate of Measurement:	2020-09-14 14:36:24.037	Date of Measurement:	2020-09-14 14:37:19.254
Capillary Type:	Unspecified container/capillary type	Capillary Type:	Unspecified container/capillary type
Capillary Position:	1	Capillary Position:	2
Ligand:	ligand description	Ligand:	ligand description
igand Concentration:	5000	Ligand Concentration:	2500
Target:	Target	Target:	Target
TargetConcentration:	n/a	TargetConcentration:	n/a
**Measurement Settings**		**Measurement Settings**	
MST-Power:	Medium	MST-Power:	Medium
Excitation-Power:	1%	Excitation-Power:	1%
Excitation type:	LabelFree	Excitation type:	LabelFree
Thermostat Setpoint:	40.0°C	Thermostat Setpoint:	40.0°C
**Included**		**Included**	
Time [s]	Raw Fluorescence [counts]	Time [s]	Raw Fluorescence [counts]
5.5141544342041	9992.29296875	-5.5141544342041	11974.638671875
5.43963861465454	9980.5830078125	-5.43963861465454	11978.1728515625
5.36512327194214	9981.939453125	-5.36512327194214	11981.4521484375
5.29060745239258	10053.0146484375	-5.29060745239258	11955.068359375
[illegible]	[illegible]	[illegible]	[illegible]

Figure 2. Example of the spreadsheet required to load the MST experiment result into ThermoAffinity.

ThermoAffinity can also load a file with no header and two columns separated by spaces, comma, or semicolon. The first column has information about the ligand concentration and the second about the signal value.

1.2. Normalization

The signal of each curve is divided by the mean value of the signal before the T-jump ($time <= 0$).

1.3. Median filter (smoothing)

The median filter consists of calculating the median value of a temperature rolling window.

1.4. Hot and cold region selection

The thermophoretic signal of the hot (F_{Hot}) and cold (F_{Cold}) regions are averaged to get the $Fnorm$ values ($\frac{F_{Hot}}{F_{Cold}}$).

2. Fitting

2.1 Model

The models implemented in ThermoAffinity are useful for all cases where a signal can be described by a linear combination of the unbound protein and complex. This can be $Fnorm = F_{hot} / F_{cold}$ (thermophoresis shift) or the initial fluorescence.

The signal is fitted using a simple model where the contribution of the complex and the unbound protein is given by the following equation:

$$Signal(K_d, L_0, P_0) = RF1 * P(K_d, L_0, P_0) + RF2 * PL(K_d, L_0, P_0) \quad (1)$$

where P and PL are respectively the unbound free protein and the bound protein. P_0 and L_0 are respectively the total protein and ligand concentration and K_d is the equilibrium dissociation constant linked to the chemical equilibrium

$$P + L \leftrightarrow PL, \ K_d = (P * L) / PL \quad (2)$$

and

$RF1$ and $RF2$ are parameters that represent the signal per unit of concentration.

Using Equation 46 and the fact that the total ligand and protein concentrations are constant, we can transform the signal to:

$$Signal(K_d, L_0, P_0) = 0.5 * ((K_d + P_0 + L_0) - \sqrt{(K_d + P_0 + L_0)^2 - 4 * P_0 L_0}) * (RF2 - RF1) + RF1 * P_0 \quad (3)$$

Two binding sites

In the case of the binding sites, the signal can be explained by a linear combination of the amount of free unbound protein, right-bound complex (PL), left-bound complex (LP), and double-bound complex (LPL).

$$Signal(L_0, P_0, K_{d,1}, K_{d,2}, cFactor) = RF1 * P + RF2 * PL + RF3 * LP + RF4 * LPL \quad (4)$$

where $RF1$, $RF2$, $RF3$ and $RF4$ are parameters to fit that represent the signal per units of concentration, L_0, P_0, $K_{d,1}$, $K_{d,2}$ and $cFactor$ are

respectively the total protein concentration, total ligand concentration, the equilibrium dissociation constant 1, the equilibrium dissociation constant 2, and cooperativity factor. The associated chemical equilibria are

$$P + 2L \Leftrightarrow PL + L \qquad (5)$$
$$P + 2L \Leftrightarrow LP + L \qquad (6)$$
$$PL + L \Leftrightarrow LPL \qquad (7)$$
$$LP + L \Leftrightarrow LPL \qquad (8)$$

with equations

$$P = K_{d,1} * K_{d,2} * (L_0 - L) /((K_{d,1} + K_{d,2} + 2 * L) * L) \qquad (9)$$
$$PL = (L * P / K_{d,2}) \qquad (10)$$
$$LP = (L * P / K_{d,1}) \qquad (11)$$
$$LPL = \frac{LP*L}{K_{d,2} * cFactor} \qquad (12)$$

where L is the free ligand concentration that corresponds to the the only physical root of the equation

$$X^3 + pX^2 + qX + r \qquad (13)$$

$$p = [K_{d,1} + K_{d,2} + (2 * P_0 - L_0) / cFactor] * cFactor \qquad (14)$$
$$q = [(P_0 - L_0) * (K_{d,1} + K_{d,2}) + K_{d,1} * K_{d,2}] * cFactor \qquad (15)$$
$$r = [- L_0 * K_{d,1} * K_{d,2}] * cFactor \qquad (16)$$

Due to the number of parameters, we have simplified this model to some alternatives.

For parameters $RF1$, $RF2$, $RF3$ and $RF4$, we have

a) $RF2 = RF3 = RF1 + \Delta F$ & $RF4 = RF1 + 2\Delta F$
b) $RF1 = RF2$ & $RF4 = RF3 = RF1 + \Delta F$

For $K_{d,1}$, $K_{d,2}$ and $cFactor$,

a) $K_{d,1} = K_{d,2}$ & $cFactor = 1$
b) $K_{d,1} = K_{d,2}$ & $cFactor \neq 1$
c) $K_{d,1} \neq K_{d,2}$ & $cFactor = 1$

2.2 Initial estimates and boundaries of the parameters

To improve the convergence of the fitting procedure, initial estimates and boundaries are estimated as follows.

Parameter	Initial value
RF2	$\frac{min(signal)}{P_0}$ if $maxLigSignal <= minLigSignal$ else $\frac{max(signal)}{P_0}$
RF1	$\frac{max(signal)}{P_0}$ if $maxLigSignal <= minLigSignal$ else $\frac{min(signal)}{P_0}$
K_d,$K_{d.1}$,$K_{d.2}$	$median(\ LigConcVec\ )$

* $maxLigSignal$ and $minLigSignal$ are respectively the signal of the position with the highest and lowest ligand (binding partner) concentration. $LigConcVec$ is the vector containing the ligand concentrations.

Parameter	Lower bound	Upper bound
RF2, RF1	$min(RF1_{Init}, RF2_{Init}) * 0.7\ if$ $min(RF1_{Init}, RF2_{Init}) > 0$ else $min(RF1_{Init}, RF2_{Init}) * 1.4$	$max(RF1_{Init}, RF2_{Init}) * 1.4\ if$ $max(RF1_{Init}, RF2_{Init}) > 0$ else $max(RF1_{Init}, RF2_{Init}) * 0.7$
Kd	$min(LigConcVec) * 1.5$	$max(LigConcVec)$ / 1.5
$K_{d.1}$,$K_{d.2}$	$min(LigConcVec) * 3$	$max(LigConcVec)$ / 3

2.3 Curve fitting

The $Fnorm$ (or initial fluorescence) versus ligand concentration is fitted using the Levenberg Marquardt algorithm. In all cases, the units of RF1 and RF2 are [1 / µM].

2.4 Fitting errors

The standard deviation of all fitted parameters is computed using the square root of diagonal values from the fit parameter covariance matrix (using the R programming language package *minpack.lm*).

Packages

ThermoAffinity is possible thanks to:

R language: R Core Team (2020). R: A language and environment for statistical computing. R Foundation for Statistical Computing, Vienna, Austria. URL https://www.R-project.org/.

R package shiny: Winston Chang, Joe Cheng, JJ Allaire, Yihui Xie and Jonathan McPherson (2020). shiny: Web Application Framework for R. R package version 1.4.0.2. https://CRAN.R-project.org/package=shiny

R package viridis: Simon Garnier (2018). viridis: Default Color Maps from 'matplotlib'. R package version 0.5.1. https://CRAN.R-project.org/package=viridis

R package tidyverse: Wickham et al., (2019). Welcome to the tidyverse. Journal of Open Source Software, 4(43), 1686, https://doi.org/10.21105/joss.01686

R package pracma: Hans W. Borchers (2019). pracma: Practical Numerical Math Functions. R package version 2.2.9. https://CRAN.R-project.org/package=pracma

R package shinydashboard: Winston Chang and Barbara Borges Ribeiro (2018). shinydashboard: Create Dashboards with 'Shiny'. R package version 0.7.1. https://CRAN.R-project.org/package=shinydashboard

R package ggplot2: H. Wickham. ggplot2: Elegant Graphics for Data Analysis. Springer-Verlag New York, 2016.

R package xlsx: Adrian Dragulescu and Cole Arendt (2020). xlsx: Read, Write, Format Excel 2007 and Excel 97/2000/XP/2003 Files. R package version 0.6.3. https://CRAN.R-project.org/package=xlsx

R package reshape2: Hadley Wickham (2007). Reshaping Data with the reshape Package. Journal of Statistical Software, 21(12), 1-20. URL http://www.jstatsoft.org/v21/i12/.

R package tippy: John Coene (2018). tippy: Add Tooltips to 'R markdown' Documents or 'Shiny' Apps. R package version 0.0.1. https://CRAN.R-project.org/package=tippy

R package shinyalert: Pretty Popup Messages (Modals) in 'Shiny'. R package version 1.1. https://CRAN.R-project.org/package=shinyalert

R package plotly: C. Sievert. Interactive Web-Based Data Visualization with R, plotly, and shiny. Chapman and Hall/CRC Florida, 2020.

R package tableHTML: Theo Boutaris, Clemens Zauchner and Dana Jomar (2019). tableHTML: A Tool to Create HTML Tables. R package version 2.0.0. https://CRAN.R-project.org/package=tableHTML

R package rhandsontable: Jonathan Owen (2018). rhandsontable: Interface to the 'Handsontable.js' Library. R package version 0.3.7. https://CRAN.R-project.org/package=rhandsontable

R package remotes: Jim Hester, Gábor Csárdi, Hadley Wickham, Winston Chang, Martin Morgan and Dan Tenenbaum (2020). remotes: R Package Installation from Remote Repositories, Including 'GitHub'. R package version 2.1.1. https://CRAN.R-project.org/package=remotes

R package devtools: Hadley Wickham, Jim Hester and Winston Chang (2020). devtools: Tools to Make Developing R Packages Easier. R package version 2.3.0. https://CRAN.R-project.org/package=devtools

R package shinyjs: Dean Attali (2020). shinyjs: Easily Improve the User Experience of Your Shiny Apps in Seconds. R package version 1.1. https://CRAN.R-project.org/package=shinyjs

R package data.table: Matt Dowle and Arun Srinivasan (2019). data.table: Extension of data.frame. R package version 1.12.8. https://CRAN.R-project.org/package=data.table

R package reticulate: Kevin Ushey, JJ Allaire and Yuan Tang (2020). reticulate: Interface to 'Python'. R package version 1.16. https://CRAN.R-project.org/package=reticulate

R package shinycssloaders: Andras Sali and Dean Attali (2020). shinycssloaders: Add CSS Loading Animations to 'shiny' Outputs. R package version 0.3. https://CRAN.R-project.org/package=shinycssloaders

R package nlstools: Florent Baty, Christian Ritz, Sandrine Charles, Martin Brutsche, Jean-Pierre Flandrois, Marie-Laure Delignette-Muller (2015). A Toolbox for Nonlinear Regression in R: The Package nlstools. Journal of Statistical Software, 66(5), 1-21. URL http://www.jstatsoft.org/v66/i05/

R package minpack.lm: Timur V. Elzhov, Katharine M. Mullen, Andrej-Nikolai Spiess and Ben Bolker (2016). minpack.lm: R Interface to the Levenberg-Marquardt Nonlinear Least-Squares Algorithm Found in MINPACK, Plus Support for Bounds. R package version 1.2-1. https://CRAN.R-project.org/package=minpack.lm

R package broom: David Robinson, Alex Hayes and Simon Couch (2020). broom: Convert Statistical Objects into Tidy Tibbles. R package version 0.7.1. https://CRAN.R-project.org/package=broom

Python3.7 language: Van Rossum, G., & Drake, F. L. (2009). Python 3 Reference Manual. Scotts Valley, CA: CreateSpace.

Python package numpy: Travis E, Oliphant. A guide to NumPy, USA: Trelgol Publishing, (2006). Stéfan van der Walt, S. Chris Colbert, and Gaël Varoquaux. The NumPy Array: A Structure for Efficient Numerical Computation, Computing in Science & Engineering, 13, 22-30 (2011), DOI:10.1109/MCSE.2011.37

Python package pandas: Wes McKinney. Data Structures for Statistical Computing in Python, Proceedings of the 9th Python in Science Conference, 51-56 (2010)

Python package scipy: Pauli Virtanen, Ralf Gommers, Travis E. Oliphant, Matt Haberland, Tyler Reddy, David Cournapeau, Evgeni Burovski, Pearu Peterson, Warren Weckesser, Jonathan Bright, Stéfan J. van der Walt, Matthew Brett, Joshua Wilson, K. Jarrod Millman, Nikolay Mayorov, Andrew R. J. Nelson, Eric Jones, Robert Kern, Eric Larson, CJ Carey, İlhan Polat, Yu Feng, Eric W. Moore, Jake VanderPlas, Denis Laxalde, Josef Perktold, Robert Cimrman, Ian Henriksen, E.A. Quintero, Charles R Harris, Anne M. Archibald, Antônio H. Ribeiro, Fabian Pedregosa, Paul van Mulbregt, and SciPy 1.0 Contributors. (2020) SciPy 1.0: Fundamental Algorithms for Scientific Computing in Python. Nature Methods, 17(3), 261-272.

Python package xlrd: https://xlrd.readthedocs.io/en/latest/index.html

Python package natsort: https://natsort.readthedocs.io/en/master/
